# Gene acquisition by giant transposons primes eukaryotes for rapid evolution via horizontal gene transfer

**DOI:** 10.1126/sciadv.adp8738

**Published:** 2024-12-06

**Authors:** Andrew S. Urquhart, Emile Gluck-Thaler, Aaron A. Vogan

**Affiliations:** ^1^ Systematic Biology, Department of Organismal Biology, University of Uppsala, Uppsala 752 36, Sweden.; ^2^ Commonwealth Scientific and Industrial Research Organisation, St. Lucia, Queensland 4067, Australia.; ^3^ Applied Biosciences, Macquarie University, Macquarie Park, New South Wales 2113, Australia.; ^4^ Laboratory of Evolutionary Genetics, University of Neuchâtel, Neuchâtel 2000, Switzerland.; ^5^ Department of Plant Pathology, University of Wisconsin-Madison, Madison, WI 53706, USA.; ^6^ Wisconsin Institute for Discovery, Madison, WI 53706, USA.

## Abstract

Horizontal gene transfer (HGT) disseminates genetic information between species and is a powerful mechanism of adaptation. Yet, we know little about its underlying drivers in eukaryotes. Giant *Starship* transposons have been implicated as agents of fungal HGT, providing an unprecedented opportunity to reveal the evolutionary parameters behind this process. Here, we characterize the *ssf* gene cluster, which contributes to formaldehyde resistance, and use it to demonstrate how mobile element evolution shapes fungal adaptation. We found that *ssf* clusters have been acquired by various distantly related *Starships*, which each exhibit multiple instances of horizontal transfer across fungal species (at least nine events, including between different taxonomic orders). Many *ssf* clusters have subsequently integrated into their host’s genome, illustrating how *Starships* shape the evolutionary trajectory of fungal hosts beyond any single transfer. Our results demonstrate the key role *Starships* play in mediating rapid and repeated adaptation via HGT, elevating the importance of mobile element evolution in eukaryotic biology.

## Teaser

Giant transposons called “*Starships*” mediate horizontal gene transfer in fungi to drive repeated evolution and rapid adaptation.

## INTRODUCTION

Long thought to be a slow process taking place over millennia, adaptive evolution is now recognized as being able to operate over much shorter timescales (*1*). Examples of rapid adaptation are now known from across the tree of life. In animals, the textbook example of rapid adaptation comes from the peppered moth, which became darker in color in response to increased pollution during the industrial revolution (*2*). Other diverse examples include marine stickleback fish that have repeatedly adapted to colonize freshwater habitats (*3*) and house finches that have evolved resistance to *Mycoplasma* infection within the past 25 years (*4*). Many organisms have rapidly evolved resistance to chemicals, which humans have used to control them. For example, fungal pathogens developed widespread resistance to the azole fungicides used in agriculture and clinical settings within several decades of their first use (*5*), bacteria have developed resistance to many antibiotics (*6*), the *Plasmodium* that cause malaria have developed resistance to antimalarials within 10 years of drugs deployment (*7*), and insects have become resistant to insecticides (*8*). There are several mechanisms by which adaptation can occur rapidly. One is that natural selection can act upon existing standing variation in the population as in the case of stickleback fish (*3*, *9*). Another is that novel mutations can create major phenotypic change in a limited number of mutational steps as is the case for fungicide resistance, which is due to simple mutations in the gene encoding the target of the fungicide (*5*, *10*, *11*). Last, an organism may evolve a new phenotype by acquiring an existing genotype in a single step from another organism, known more simply as horizontal gene transfer (HGT) (*12*). This last mode is well established as a driving force in the emergence of bacterial antibiotic resistance (*13*) but is poorly understood as a mechanism in eukaryotic evolution.

Despite generally lacking identified mechanisms enabling HGT in eukaryotes, there are a growing number of eukaryote-to-eukaryote HGTs implicated in adaptation. For example, the whitefly, *Bemisia tabaci*, obtained a gene from plants required to detoxify phenolic glucosides (*14*). Similarly, aphids obtained the genes for carotenoid production from fungi (*15*). In fish, an antifreeze protein gene horizontally transferred from herring to smelt (*16*, *17*). HGT seems to be particularly important in fungi. By the year 2000, there was growing, albeit inconclusive, evidence for fungal to fungal HGT, largely in the form of discordance between gene and species phylogenies (*18*). More concrete evidence came by 2006 with the discovery that the gene encoding the plant host-specific toxin, *ToxA*, is 99.7% identical between *Pyrenophora tritici-repentis* and *Parastagonospora nodorum* (*19*). This similarity was difficult to explain in the absence of HGT. Since then, numerous additional examples of HGT have emerged including adaptation to cheese among fungi used in making cheese and numerous metabolic gene clusters (*20*–*25*). However, we know little about the underlying drivers of eukaryotic HGT because its mechanisms are not yet well understood. A better understanding of the genetic mechanisms underlying rapid evolution, including HGT, is of fundamental importance as it will help us predict if and when adaptive phenotypes might emerge. In fungi, this is a major problem as they threaten both human health and food security (*26*, *27*).

*Starships* are a recently described superfamily of massive fungal transposons, which mobilize DNA within and between fungi (*28*, *29*). Similar to other fungal transposons, *Starships* are sequences embedded within genomes that are capable of movement, i.e., transposition from one genetic locus to another. In the case of *Starships*, this movement is mediated by a tyrosine recombinase (YR, termed the “captain”) that is always present as the first gene of the element. The key difference between *Starships* and other fungal transposons is their much larger size, which can be up to 700 kb, but more typically around 100 kb. The large size of *Starships* is because in addition to carrying the captain recombinase required for transposition, they carry huge amounts of additional gene cargo (tens to hundreds of genes).

Some of the genetic cargo carried by *Starships* is adaptive for their fungal host. For example, the *Hephaestus* (*H*φ) *Starship* of *Paecilomyces variotii* provides resistance to at least five toxic metal ions, namely, zinc, cadmium, copper, lead, and arsenic (*30*). The *Starships Horizon* and *Icarus* of the wheat pathogen *P. tritici-repentis* contain the *ToxA* and *ToxB* genes, respectively, which increase pathogenicity on wheat (*31*). Similarly, *Starship Voyager* is associated with a complex trade-off between saprotrophy and pathogenicity in the broad host range pathogen *Macrophomina phaseolina* (*28*). Even the population-level frequencies of the *Starship Swordfish*, which is no longer presumed to be active, are associated with changes in climatic conditions in the wheat pathogen *Zymoseptoria* (*32*). In addition, many of these *Starships*, and/or their cargo, display signatures of HGT, implicating these massive mobile elements in the process of rapid adaptation. Furthermore, a much broader, yet uncharacterized, set of gene content is present in other predicted *Starships*, suggesting that a vast diversity of fungal phenotypes are mediated through HGT (*29*, *33*).

Here, we illuminate the role that *Starships* play as drivers of rapid adaptation in fungi by investigating the evolution and function of the putative formaldehyde detoxification gene cluster, *ssf*. We first found that *ssf* clusters have been acquired by four *Starships* belonging to distantly related families. Through a nucleotide similarity-based approach, we show that each of the four elements containing the *ssf* cluster have horizontally transferred between species. We demonstrate that HGT of the *Starship Chrysaor* (*Χ*ρ) enabled rapid adaptation to formaldehyde in both *Aspergillus fumigatus* and *P. variotii* and confirm that this adaptation is mediated by the *ssf* cluster through gene knockouts. Phylogenetic analyses confirm a single origin of the *ssf* cluster and its association to *Starships*, implicating intermobile element genetic exchanges as a driver for HGT. We also observe that a number of *ssf* clusters have integrated into genomic regions that are no longer associated with *Starship* elements, suggesting that the birth-and-death processes of giant transposons shape the distributions of genes in fungal genomes beyond the life span of any single element. We argue that the *ssf* cluster is not unique and that any genes or gene clusters, which provide a similar selective advantage, could be transferred by *Starships* and subsequently be retained in a new host species. The spread of the *ssf* cluster across different *Starships* and fungal species additionally provides key evidence supporting the predictions of the decades-old “selfish gene cluster” and “selfish operon” hypotheses that postulate that HGT selects for the clustering of metabolic genes, implicating *Starships* in the formation and evolution of fungal gene clusters (*34*, *35*). Thus, our results reveal that *Starship* dynamics drive the rapid emergence of adaptive fungal phenotypes and elevate HGT from a phenomenon that rarely affects eukaryotic genomes to an impelling evolutionary force.

## RESULTS

### The *ssf* region putatively responsible for formaldehyde resistance has been horizontally transferred by four different *Starship*s

**Fig. 1.**
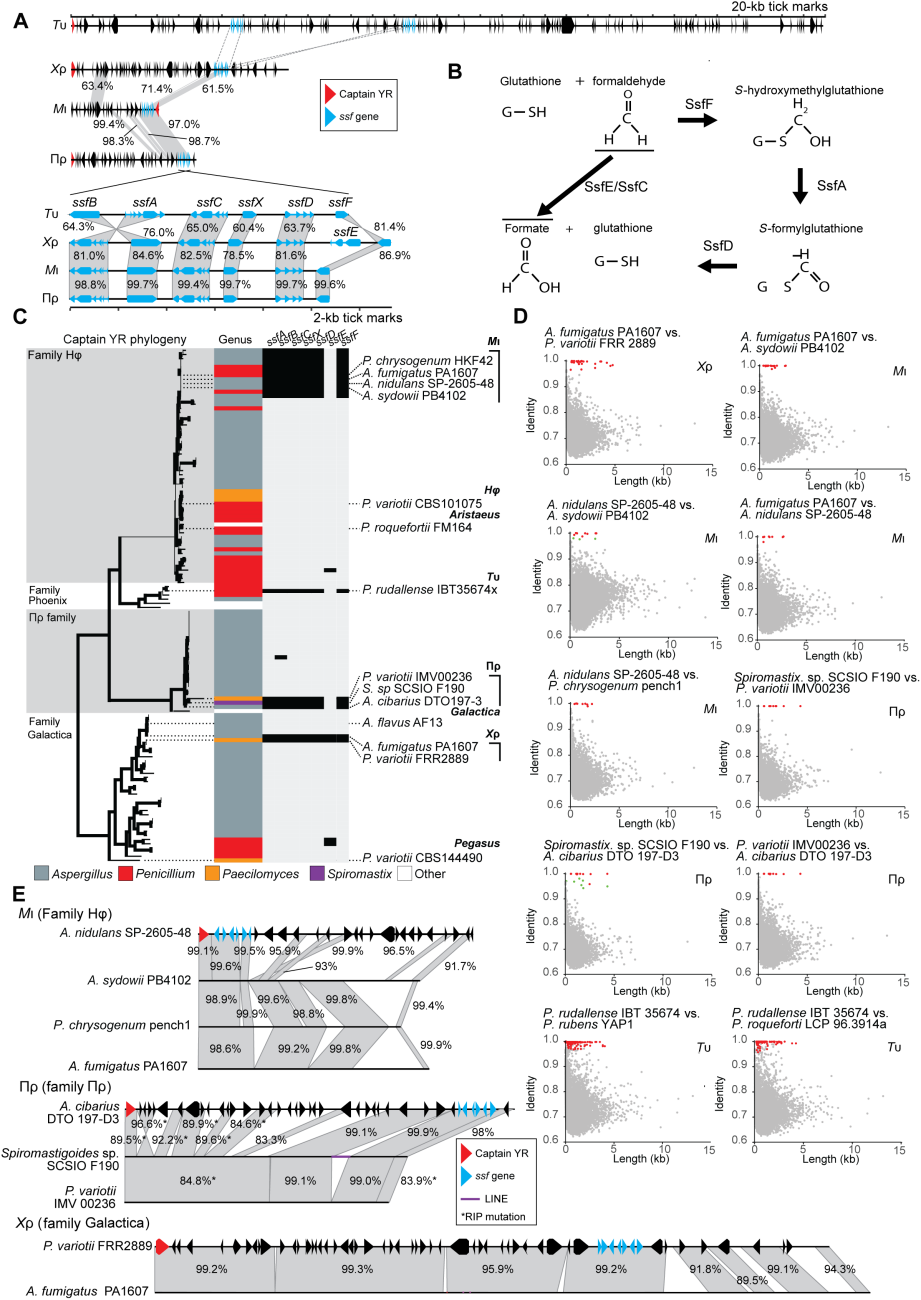
The *ssf* region putatively involved in formaldehyde detoxification has been horizontally transferred on four different *Starship* families. (**A**) Representative from each family of *ssf Starships* showing the position of the *ssf* gene region and below a comparison of the *ssf* region found in each *Starship*. The two copies of the *ssf* region in *T*υ are nearly identical, so only one of these is shown. Nucleotide identity is given in percentages based on Mauve alignments (*105*). Dashed lines between *T*υ and *Χ*ρ indicate homologous *ssf* regions too divergent to be aligned by Mauve. (**B**) Predicted roles of *ssf* genes in the glutathione-dependent formaldehyde detoxification pathway. (**C**) A phylogeny of *Starship* captain proteins from elements with the putative *ssf* cluster homologs and close relatives. Note that *A. fumigatus* strain PA1607 contains both *M*ι and *Χ*ρ. (**D**) BLAST-all genome comparisons between genomes containing copies of the *ssf Starships*. Each point represents the best BLAST result from a single-gene sequence. Points highlighted in red are genes within *Starships* that we consider to represent HGT based on high-sequence identity in these genes compared to the remaining genes in the genome. Points highlighted in green are external to the *Starship* in one of the genomes. (**E**) Comparison of identified *ssf Starships* within each family reveals large amounts of highly similar DNA in each case. Nucleotide identity is given in percentages based on Mauve alignments (*105*). RIP, repeat-induced point.

We previously identified the *Starship Mithridate* (*M*ι) from the Hephaestus-family as a *Starship* carrying a gene cluster encoding a number of proteins homologous to known formaldehyde-detoxifying enzymes (*29*). We name this region the *Starship* formaldehyde cluster (*ssf*) (Fig. 1A). The *ssf* region contains at least six and up to seven neighboring genes, the predicted functions of which are listed in Table 1. At least three of these were predicted to encode components of the glutathione-dependent formaldehyde detoxification pathway, namely, *ssfF*, *ssfA* and *ssfD* (Fig. 1B) (*36*–*39*).

**Table 1.** Predicted function of genes found within the *ssf* cluster.

Gene	Predicted function/homology
*ssfF*	SsfF shows similarity to the formaldehyde-activating enzyme (Gfa) from *Paracoccus,* which is believed to increase the rate of reaction between fomaldehyde and glutathione to form *S*-hydroxymethylglutathione (*55*).
*ssfA*	SsfA shows 92% amino acid identity to the characterized *S*-hydroxymethylglutathione dehydrogenase of *Paecilomyces* sp. no. 5 FldA, which was found to functionally complement a *Saccharomyces cerevisiae* glutathione-dependent formaldehyde dehydrogenase mutant. As noted in the original genome announcement, a formaldehyde gene cluster is also found in this species, and *fldA* is part of this cluster (*57*, *77*, *107*).
*ssfB*	Not homologous to known formaldehyde detoxifying enzymes.
*ssfX*	Not homologous to known formaldehyde detoxifying enzymes.
*ssfC* and *ssfE*	SsfC and SsfE both show homology to alcohol dehydrogenase enzymes. In particular, SsfC shows weak similarity to characterized alcohol dehydrogenase class 3 homologs, the closest of which is the *Pseudomonas putida* formaldehyde dehydrogenase fdhA, which is independent of glutathione. SsfE shows 82% amino acid identity to *Aspergillus oryzae* alcohol dehydrogenase AlcA, which when deleted reduces the ability of this fungus to degrade formaldehyde, although the mechanisms are unclear (*108*–*110*).
*ssfD*	*ssfD* encodes an *S*-formylglutathione hydrolase. This enzyme converts *S*-formylglutathione to formate and glutathione (*107*).

We have hypothesized that *Starships* disseminate traits that are beneficial for their genomic hosts because this might serve to increase the *Starship’s* own chances of reproductive success (*29*). We therefore examined the role of *Starships* in the horizontal movement of adaptive phenotypes using the *ssf* resistance cluster as a test case. We searched a large database of previously identified *Starships* for additional elements containing the *ssf* cluster (*40*). This revealed that the *ssf* cluster is present in four distinct families of *Starships*: *Mithridate* [*M*ι, Hephaestus-family (*30*)], *Prometheus* [Πρ, Prometheus-family (*40*)], *Chrysaor* [*Χ*ρ, Galactica-family (*28*)], and *Typhon* [*T*υ, Phoenix-family (*28*)] (Fig. 1C).

Given that we previously showed that *M*ι likely horizontally transferred (not necessarily directly) between *Aspergillus* and *Penicillium* (*29*), we decided to investigate additional instances of horizontal transfer involving each of the families of *ssf*-carrying *Starships*. To do this, we used a “BLAST-all” approach that we have used previously to identify coding sequences with unusually high-sequence similarity between two genomes (*29*, *30*, *41*) (Fig. 1D). For *M*ι, in addition to being found within the genomes of *Aspergillus nidulans* SP-2605-48 (*42*) and *P. chrysogenum* “pench1,” as previously reported (*30*), *M*ι was also found within the genomes of several species in *Aspergillus* subsection *Versicolores* and *A. fumigatus* PA1607. From subsection *Versicolores*, we selected a representative *M*ι from “*Aspergillus* sp.” PB4102, which we taxonomically identified as *Aspergillus sydowii* based on a phylogenetic comparison of the *RPB2* sequence to those examined by Jurjevic *et al.* (*43*) (fig. S1). BLAST-all comparisons between these four *M*ι-containing strains revealed a high level of sequence identity within *M*ι, which is inconsistent with vertical descent from a common ancestor (Fig. 1, D and E). For example, BLAST of the *A. fumigatus* PA1607 gene regions versus the *A. sydowii* PB4102 assembly contigs returned hits for 7652 genes with 20 genes returning a hit >100 base pair (bp) and >96% identify (highlighted in red in Fig. 1D). Of these 20 hits, all were from genes located in the *A. fumigatus M*ι region (representing all of the *M*ι genes predicted). In contrast, the remaining genes had BLAST hits with an average identity of 72.2%. Thus, at least two HGT events must have occurred between these pairs of *Aspergillus* species in addition to the HGT event reported previously, for a total of four events.

Πρ belongs to a recently described family of *Starships*, which targets AT-rich sites (*40*). We have identified three copies of Πρ containing the *ssf* cluster found within *Spiromastigoides* [previously *Spiromatix* (*44*)] sp. SCSIO F190 (*45*), *P. variotii* IMV00236 (*46*), and *Aspergillus cibarius* DTO 197-D3 (Fig. 1E). *Spiromastix* belongs to the order Onygenales and the latter two species to the order Eurotiales. Both the *P. variotii* and *Spiromastigoides* sp. Πρ are heavily affected by repeat-induced point (RIP) mutations, which are a fungal genome defense system that mutates repetitive DNA in the genome (*47*), and we thus consider these to represent immobile “derelict” *Starships*. Nevertheless, pairwise BLAST-all comparisons between these species showed highly conserved gene content within the *Spiromastigoides* sp. region in each species (Fig. 1D). For example, BLAST-all comparison between the *Spiromastigoides*. sp. SCSIO F190 gene regions versus the *P. variotii* IMV00236 contigs returned a hit for 6176 genes with 10 genes returning a hit >100 bp and 100% identify (highlighted in red in Fig. 1D). Of these 10 hits, all were located in the *Spiromastigoides *Πρ region (of 18 Πρ genes predicted). In contrast, the hits in the rest of the genome showed only 72.3% average nucleotide identity. HGT of the *ssf* cluster must have occurred at least twice on Πρ including between the Eurotiomycete orders Onygenales and Eurotiales.

*T*υ is a newly found *Starship* of the Phoenix-family (*28*), for which only one fully assembled element carrying the *ssf* cluster has been found. This is a ~700-kb *Starship* in the genome of “*Pencillium* sp.” strain IBT 35674x, which we identified as *A. rudallense* based on comparison of the RPB2 sequence to those examined by Houbraken *et al.* (*48*) (fig. S1). The massive size of the element, the largest *Starship* so far identified, means it is likely to be intact only in highly contiguous assemblies. However, at least two other *Penicillium* genomes contain a fragmented *T*υ, *Penicillium rubens* YAP1 and *P. roqueforti* LCP 96.3914a [genome published in (*49*)]. BLAST comparison between *P. rudallense* and these genomes revealed signals for HGT both within and outside of the identified *Tυ Starship* (defined arbitrarily as genes with BLASTn matches with >100 bp length and >97% identity). The highly conserved genes found outside of the identified *Tυ Starship* are found within a second *T*υ element (which we designate *T*υ haplotype 2) and on contig 8 where a degraded relict of *T*υ is found (Fig. 1D and fig. S2). Given the presence of a derelict *Starship*, we believe that the highly similar DNA on chromosome 8 represents DNA derived from this degrading *Starship*. Thus, HGT of the *ssf* cluster must have occurred at least twice on *T*υ between *Penicillium* species.

Lastly, in addition to being found within *P. variotii*, *Χ*ρ is found within certain *A. fumigatus* strains (including PA1607, which also contains *M*ι), and this represents another HGT event (Fig. 1, D and E). BLAST searching *A. fumigatus* PA1607 gene regions as queries against the *P. variotii* FRR 2889 assembly contigs returned BLAST hits for 6995 genes. Of these, 29 hits were more than >100 bp in length and more than 96% nucleotide identity (highlighted in red in Fig. 1D). All of these hits were located within the *Χ*ρ region, in which 35 genes were identified in the copy found in PA1607. In contrast, the average nucleotide identity in the remaining BLAST hits was 72.5%. BLASTn searches also revealed the presence of *Χ*ρ in the genome of *Aspergillus flavus* strain B7001B, and while this assembly is highly fragmented, BLAST-all comparison between this genome and *A. fumigatus* PA1607 revealed a strong HGT signal within *Χ*ρ (fig. S3).

In addition to looking at *Starships* carrying genes associated with formaldehyde tolerance, we have also identified an additional HGT of the original metal resistance *H*φ-*Starship* via BLAST searches in the recently released genome of *Penicillium chermesinum* IBT 19713 (fig. S4) (*50*). This *Penicillium Starship* is nearly identical to the original *P. variotii Starship* except for the insertion of a retrotransposon (99.8% identical). The small amount of variation is mostly single-nucleotide indels. Given that the *P. chermesinum* genome was generated using nanopore technology without additional polishing using Illumina reads, indel sequencing errors are expected and may account for the small amount of divergence observed (*50*).

### The transfer of a *Starship* carrying the *ssf* region underlies the evolution of formaldehyde resistance in both *P. variotii* and *A. fumigatus*

We hypothesized that the observed horizontal transfer of multiple *Starships* containing the *ssf* region would enable the repeated evolution of formaldehyde resistance phenotypes in evolutionarily distinct lineages. To test this hypothesis, we focused on characterizing *Starship Χ*ρ because it contains all seven *ssf* genes, and it is found in *A. fumigatus* and *P. variotii.* These two species are experimentally amenable with relatively large population-level sequencing datasets and represent both the putative donor (*Aspergillus*) and recipient (*Paecilomyces*) lineages of a *Starship-*mediated HGT event of the *ssf* cluster.

**Fig. 2.**
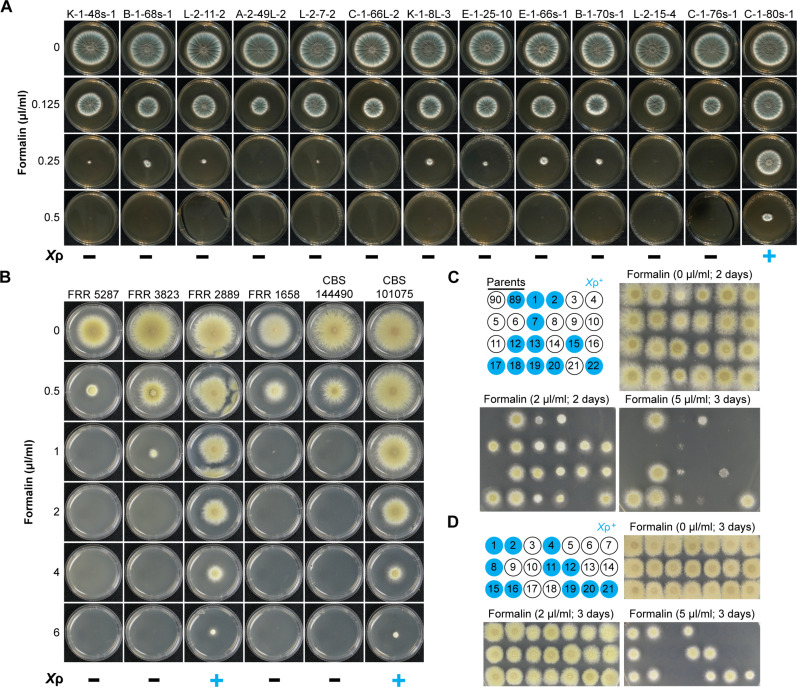
*Χ*ρ confers resistance to formaldehyde in *P. variotii* and *A. fumigatus*. (**A**) Wild-type *A. fumigatus* strains grown on varying formaldehyde concentrations showing increased formaldehyde resistance in the *Χ*ρ^+^ strain C-1-80s-1. (**B**) Six *Paecilomyces* strains grown on varying formaldehyde concentrations. The two strains FRR 2889 and CBS 10175 containing *Χ*ρ are more resistant to formaldehyde than the four wild-type strains in which the region is absent. Plates were cultured at 37°C for 2 days. (**C**) Genetic segregation shows that *Χ*ρ contributes to increased formaldehyde tolerance in strains FRR 2889 and CBS 101075. Cosegregation was not apparent in formalin (2 μl/ml), whereas in formalin (5 μl/ml), growth did cosegregate with *Χ*ρ. However, there was large variation, with progeny #1 particularly germinating but showing poor growth, which is barely visible. Those progeny that do not inherit *Χ*ρ show zero growth. (**D**) Back cross of progeny #6 from (D) to the *Χ*ρ^+^ parental strain FRR 2889 produced progeny that all grew on formalin (2 μl/ml and 5 μl/ml), segregated with the presence of Χρ. Plates were cultured at 30°C for 3 days.

We first investigated a larger database of 512 publicly available short read archive datasets of *A. fumigatus* for the presence of *Χ*ρ (*51*, *52*). We found evidence for a full, nontruncated copy in just one strain and subsequently were able to acquire this strain and 12 others that had no evidence of *Χ*ρ (fig. S5). Testing the formaldehyde tolerance of these *A. fumigatus* strains revealed that the strains lacking *Χ*ρ were unable to grow in 0.5 μl/ml formalin and showed either zero or restricted growth in 0.25 μl/ml formalin. In contrast, the *Χ*ρ^+^ strain C-1-80s-1 grew relatively well in 0.25 μl/ml formalin and continued to show some growth in 0.5 μl/ml formalin (Fig. 2A). We then assessed the distribution of *Χ*ρ within a set of six *Paecilomyces* strains available in our laboratory. These were five strains of *P. variotii* and one strain, FRR 5287, which belongs to its closest relative, *P. paravariotii* (*41*). We found that *Χ*ρ is present only in *P. variotii* FRR 2889 and as a truncated copy in CBS 101075 (fig. S5). Testing the formaldehyde resistance of these strains revealed that the two *Paecilomyces* strains containing *Χ*ρ were more resistant than the four lacking *Χ*ρ (Fig. 2B). The *Χ*ρ^+^ strains both showed growth in 6 μl/ml formalin, whereas the *Χ*ρ^+^ strains were all unable to grow in 2 μl/ml formalin. Together, these results demonstrate that the contribution of *Χ*ρ to formaldehyde resistance is maintained across HGT events.

To support the hypothesis based on this correlation that *Starship Χ*ρ is indeed responsible for the observed increase in formaldehyde resistance, we checked segregation patterns in *Paecilomyces* genetic crosses by phenotyping progeny in the presence of formaldehyde and genotyping the progeny by polymerase chain reaction (PCR) to assess the presence or absence of the formaldehyde region. Unexpectedly, segregation analysis using the progeny of a cross between FRR 2889 (*Χ*ρ^+^) and CBS 144490 (*Χ*ρ^−^) revealed that *Χ*ρ is only partially responsible for the increased formaldehyde resistance phenotype of these two isolates (Fig. 2C). In formalin (2 μl/ml; a concentration at which the *Χ*ρ^−^ isolates do not germinate), 18 of the 22 progeny were able grow, i.e., close to 75% instead of the expected 50%, which suggested that formaldehyde resistance might be linked to more than one independently segregating loci. The four progeny unable to germinate were all *Χ*ρ^−^. Increasing formalin to 5 μl/ml resulted in perfect segregation with the 11 *Χ*ρ^+^ progeny all germinating. However, the degree of growth varied widely with some *Χ*ρ^+^ progeny germinating but then showing very restricted growth (Fig. 2C). Backcrossing progeny #6, which is *Χ*ρ^−^ but presumably has the unmapped formaldehyde resistance loci given that it is able to grow at 2 μl/ml, to the *Χ*ρ^+^ parental strain FRR 2889 results in progeny that all grow at 2 μl/ml and in which the presence of *Χ*ρ cosegregates with strong growth in formalin (5 μl/ml) (Fig. 2D). This indicated that at least one unmapped locus, together with *Χ*ρ, is contributing to increased formaldehyde resistance in these isolates.

### The *ssfB* gene makes the greatest contribution to formaldehyde tolerance

**Fig. 3.**
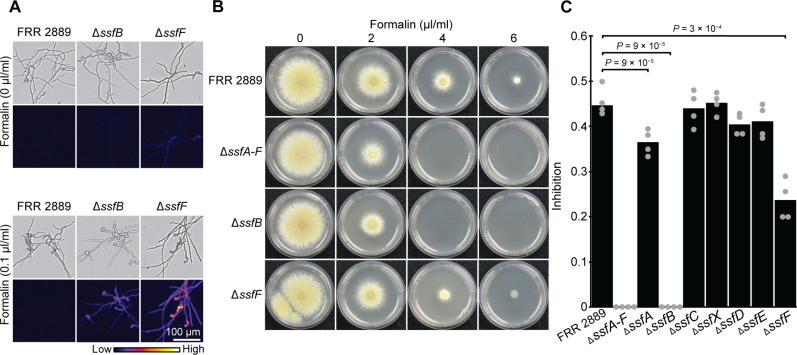
The *ssfA-F* subregion confers resistance to formaldehyde. (**A**) GFP replacement strains indicated that some *ssf* genes, including *ssfB* and *ssfF*, were strongly induced in response to formaldehyde [formalin (0.1 μl/ml)], a complete gene set is shown in fig. S6. (**B** and **C**) Deletion of the entire *ssf* cluster, Δ*ssfA-F*, resulted in decreased formaldehyde resistance comparable to deletion of a single-gene *ssfB*. The Δ*ssfF* mutant showed a milder phenotype characterized by decreased growth rate in formalin (4 μl/ml). Plates were cultured at 30°C for 3 days. Inhibition in (C) was calculated by dividing radial growth rate on CV8 plates after 3 days at 30°C in the presence of formalin (4 μl/ml) by radial growth rate in the absence of formalin. Colony diameters used to calculate inhibition are given in table S1.

We next investigated the genetic basis of *ssf*-mediated formaldehyde resistance and found that genes within this cluster contribute unequally to this phenotype. First, the coding region of each individual *ssf* gene was replaced with that of green fluorescent protein (GFP) such that GFP expression came under the control of each native *ssf* promoter, thus allowing induction in response to formaldehyde to be visualized. Some induction was present in *ssfA*, *ssfB*, *ssfC*, *ssfD*, and *ssfF* after the strains were germinated overnight in liquid media containing formalin (0.1 μl/ml) with the greatest induction observed in *ssfA*, *ssfB*, and *ssfF* (Fig. 3A and fig. S6). The response of native *ssf* promoters to formaldehyde supports a role for this region in formaldehyde tolerance.

We then proceeded to examine the formaldehyde tolerance of a strain in which the entire *ssfA-F* region was replaced by GFP under the promoter of the *ssfA* gene (Δ*Χ*ρ^+^ Δ*ssfA-F*) (Fig. 3, B and C, and table S1). The Δ*Χ*ρ^+^ Δ*ssfA-F* strain showed the expected increased sensitivity to formaldehyde with a lack of growth in formalin (4 μl/ml) (Fig. 3, B and C). Consistent with the segregation analysis, tolerance in this strain remained higher than the *Χ*ρ^−^ wild-type strains with the ability to grow in formalin (2 μl/ml) being retained in the mutant. This is congruent with the hypothesis that a second unlinked locus in FRR 2889 and CBS 101075 contributes to increased formaldehyde tolerance in these two *Χ*ρ^+^ wild-type strains. To confirm the presence of at least one additional formaldehyde resistance locus in the genome, we crossed this *Χ*ρ^+^ Δ*ssfA-F* mutant to the *Χ*ρ^−^ wild-type CBS 144490. Among 19 progeny, 14 were able to grow in formalin (2 μl/ml) and none in formalin (4 μl/ml). Resistance to formalin (2 μl/ml) did not cosegregate with the presence of *Χ*ρ^+^, which was determined by hygromycin resistance that was introduced at the same time as GFP to select transformants (fig. S7). We did not identify the genetic bases for the difference in background resistance between *Paecilomyces* strains.

Having shown that the *ssfA-F* subregion was responsible for formaldehyde tolerance, we next investigated the individual contributions of each *ssf* gene (Fig. 3, B and C, and fig. S8). Examination of the formaldehyde resistance of the single gene GFP replacement mutants revealed greatly reduced growth in the *ssfB* mutant with a complete absence of growth in formalin (4 μl/ml). This suggested that *ssfB* is a major contributor to the increased formaldehyde resistance associated with the *ssfA-F* subregion despite this gene having no clear role in formaldehyde tolerance based on characterized protein homologs. The biological role of *ssfB*, a disulfide oxidoreductase, is unclear; however, one group of disulfide reductases, namely, glutathione reductases, reduce glutathione disulfide (GSSG) to glutathione (GSH), which maintain the oxidative balance of the cell. The reaction of glutathione and formaldehyde has been shown to alter the GSH:GSSG ratio and trigger redox stress in human cells and nematodes (*53*, *54*). Hence, a role for *ssfB* in maintaining reduced glutathione, or redox balance more generally, is possible. The identification of this enzyme is a promising lead for further biochemical investigation. *ssfF* had a more subtle effect on formaldehyde tolerance as assessed by radial growth rate in formalin (4 μl/ml). *ssfF* shows similarity to the formaldehyde-activating enzyme (Gfa) from the bacteria *Paracoccus*, which is believed to increase the rate of reaction between formaldehyde and glutathione to form *S*-hydroxymethylglutathione (*55*); however, a more recent study suggests that Gfa does not directly catalyze this reaction (*56*). Previous studies have not linked a formaldehyde-activating enzyme to a phenotype in any organism. Complementation of the *ssfB* and *ssfF* mutants with wild-type copies of the deleted genes restored formaldehyde resistance to near wild-type levels (fig. S9 and table S2). Deletion of the remaining *ssf* genes did not have a major impact of formaldehyde resistance under the conditions tested (Fig. 3C).

### *ssfD* and *ssfF* are functionally redundant with their paralogs in the host genome

**Fig. 4.**
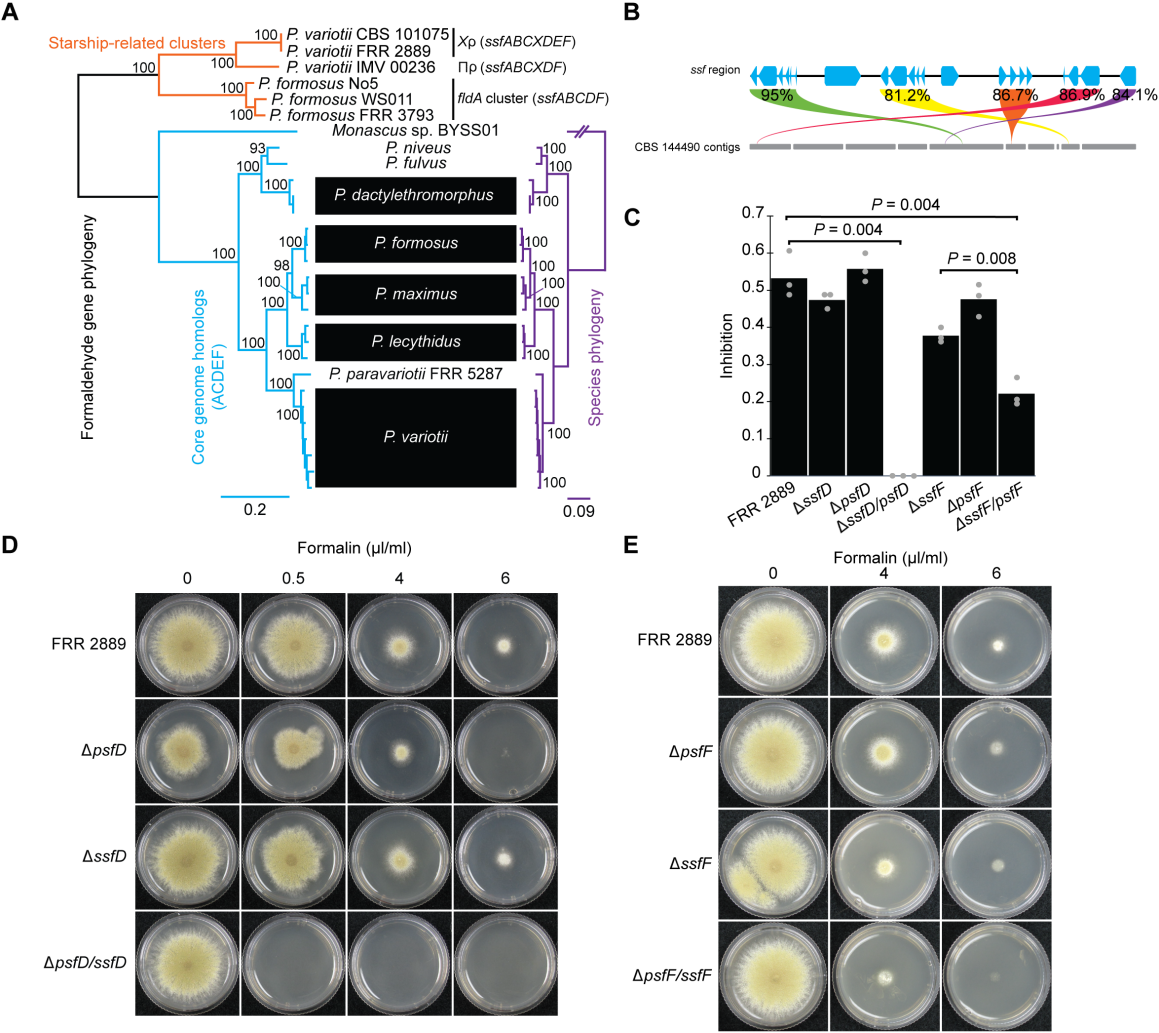
Some *ssf* genes are at least partially functionally redundant with the corresponding host homologs. (**A**) Phylogeny of formaldehyde resistance genes (concatenated gene regions) in *Paecilomyces* strains compared to species relationships. Tree was generated in IQ-TREE with branch supports calculated from 10,000 ultrafast bootstraps (*101*, *102*). The species phylogeny is taken from Urquhart and Idnurm (*41*). (**B**) Similarity (BLOSUM62 (*106*) between the Ssf proteins and their closest paralog in the *Χ*ρ^−^ strain CBS 144490. (**C** and **D**) Growth rate of Δ*ssfD*, Δ*psfD*, and the Δ*ssfD/psfD* double mutant on formaldehyde-containing media. [(C) and **E**] Growth rate of the Δ*ssfF*, Δ*psfF*, and the Δ*ssfF/psfF* double mutant on formaldehyde-containing media. Plates were cultured at 30°C for 3 days. Inhibition (C) was calculated by dividing radial growth rate on CV8 plates after 3 days at 30°C in the presence of formalin (4 μl/ml) by radial growth rate in the absence of formalin. Colony diameters used to calculate inhibition are given in table S3.

In addition to the *ssf* genes within *Χ*ρ, *Paecilomyces* genomes contain additional genes with putative roles in formaldehyde resistance, which are distributed across presumably nonmobile regions of the genome. For example, the *P. variotii* genome has homologs of *ssfA*, *ssfC*, *ssfD*, *ssfE*, and *ssfF*, but not *ssfB* and *ssfX* (Fig. 4, A and B). These “host paralogs” appear to be vertically inherited, as their phylogenetic relationships within the *Paecilomyces* genus match established species relationships (Fig. 4A). The host paralogs form a distinct monophyletic group to the exclusion of the clade of *Starship-*related *ssf* homologs. We hypothesized that some of the *ssf* genes might be partially or fully redundant with their corresponding host paralogs. Although the single gene knockout that produced the strongest phenotype was *ssfB*, this gene lacks a clear paralog in *P. variotii*. Therefore, we chose to test this hypothesis by deleting the paralogs of *ssfD* and *ssfF*, which we named *psfD* and *psfF*, respectively (paralog of *ssf*). We chose *ssfD* because this gene has a putative role in the conversion of *S*-formylglutathione to formate + glutathione (Fig. 1B). We anticipated that an *ssfD/psfD* double mutant would be highly impaired in formaldehyde resistance. Single deletions of *ssfD* or *psfD* had minimal impact on formaldehyde resistance. However, an Δ*ssfB/psfD* double mutant had increased sensitivity to formaldehyde with minimal growth even in formalin (0.5 μl/ml) (Fig. 4, C and E, and table S3). This suggests that these two paralogs are functionally redundant and do not contribute additively to formaldehyde resistance under test conditions.

On the other hand, we chose the gene *ssfF* based on its biological novelty as it has only been examined in prokaryotes and has never been experimentally linked to formaldehyde metabolism in eukaryotes. An Δ*ssfF/psfF* double mutant showed decreased growth relative to wild type in formalin plates (4 μl/ml), while the single gene knockouts showed either no (*psfF*) or modest (*ssfF*) reduction in growth rate (Fig. 4, C and F, and table S3). These results again suggest functional redundancy between host and *Starship* paralogs under the examined experimental conditions. The less notable effect of *ssfF/psfF* deletion compared to *ssfD*/*psfD* deletion is consistent with the fact that the reaction of formaldehyde and glutathione can be a spontaneous process, which does not depend on enzymatic activity (*55*).

### The *ssf* regions carried as *Starship* cargo illustrate birth-and-death processes with evidence of inter-*Starship* gene exchange and eventual incorporation of cargo into the host genome

**Fig. 5.**
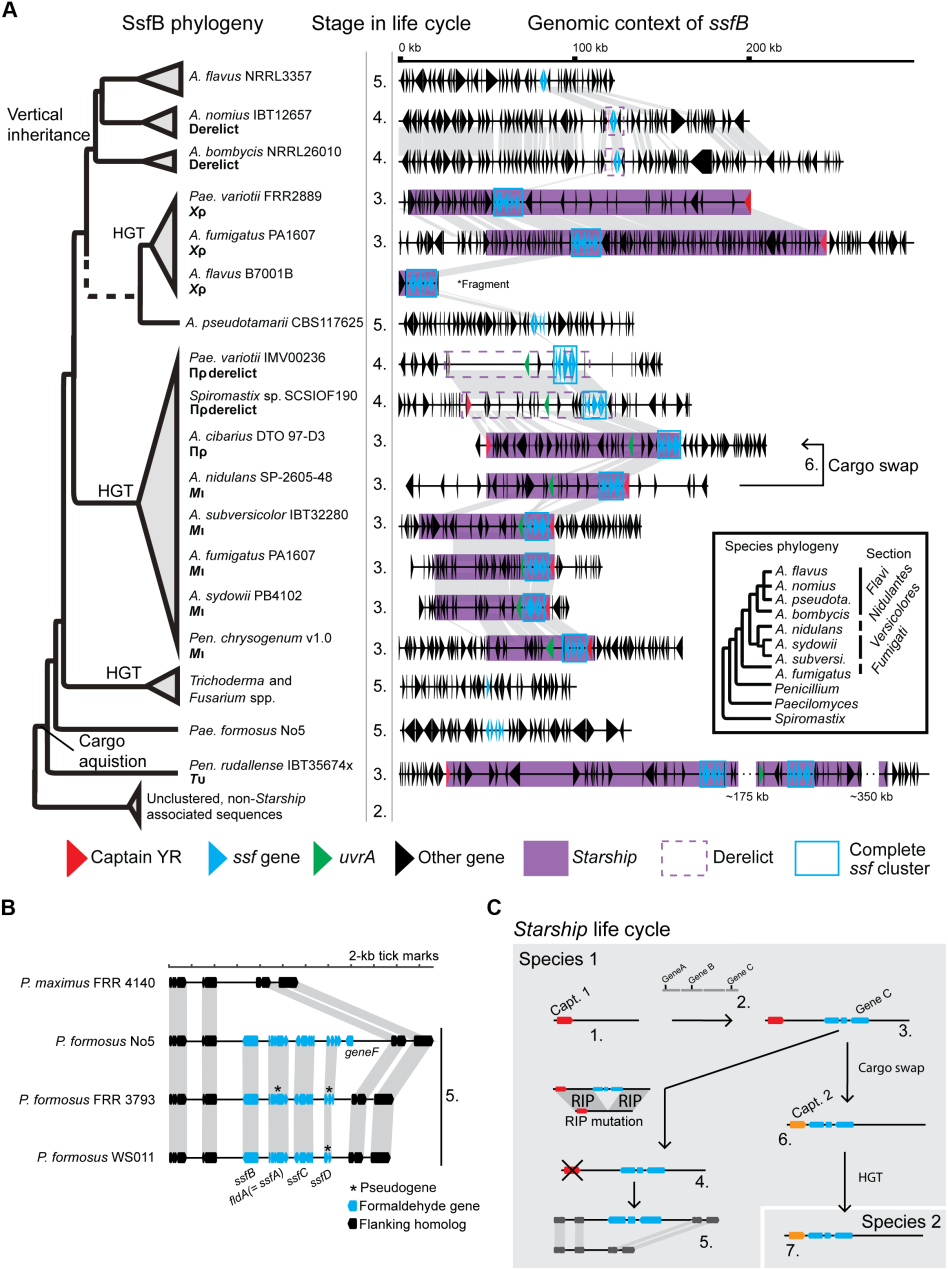
*ssf* clusters have moved both between *Starships* and into normal genomic regions. (**A**) SsfB protein phylogeny with corresponding *ssf* cluster containing regions is illustrated. The dashed line indicates a poorly supported branch (0.1% SH-aLRT and 57% UFboot support). “Cargo acquisition” refers to the original acquisition of the *ssf* cluster genes by a *Starship*. “Vertical inheritance” refers to a loss of *Starship* features followed by vertical inheritance of the *ssf* cluster. Numbers on the right hand side correspond to stages in the *Starship* life cycle shown in (C). (**B**) *ssf* cluster in *P. formosus*, which is homologous to the *ssf* cluster but is not associated with *Starship* features, appears to represent an insertion into the genome, which is degraded to varying extents in three different *P. formosus* strains. “5.” refers to step 5 in (C), which is the step in the *Starship* life cycle that we believe this cluster represents. (**C**) Diagram illustrating the proposed “life cycle” of a *Starship*. (1) An ancestral *Starship* not containing the cluster. “Captain” refers to the gene encoding the captain recombinase, which is responsible for *Starship* mobility. (2) Unlinked genes within the genome. (3) A *Starship* in which the genes have become linked and are now present as a cluster. (4) A derelict *Starship*, which has been mutated and is now immobilized. RIP mutation refers to repeat induced point mutation. (5) Genes originally carried into the genome by a *Starship* now appear as an indel relative to a sister species. (6) A cargo swapping between *Starships* has moved the gene cluster into a different *Starship*. (7) Acquisition of the gene cluster provides an adaptive advantage to the strain carrying the *Starship*, which promotes horizontal transfer into a different fungal species. An HGT event of the cluster-carrying *Starship* (3) could also occur.

To better understand the evolutionary history of the *ssf* cluster, we searched 2899 publicly available fungal genomes sampled from across the fungal tree of life (table S1) and generated individual phylogenies of *ssfB*, *ssfD*, *ssfX*, and *ssfF*. All of the gene trees exhibit topologies that are incongruent with species relationships (Fig. 5A, figs. S10 to S13, and table S4). We used these data to search for genomic neighborhoods containing clusters of *ssf* genes defined by having hits to at least three different genes within 25,000-bp distance of each other. There are remarkably few instances that fit these criteria across the fungal kingdom, consistent with these genes generally being unclustered (table S2). We used the evolutionary history of *ssfB* as a proxy to understand the history of the *ssf* cluster and its associated phenotypes, as it makes the greatest contribution to formaldehyde resistance and is consistently present across all *Starship*-associated *ssf* clusters. All *ssfB* sequences form a supported monophyletic clade with the *T*υ sequences sister to the other sequences from the other three *Starships*, consistent with the lower pairwise similarity of its *ssf* cluster to the others (Fig. 1A). *M*ι and Πρ sequences form a single clade in agreement with a very recent exchange of the *ssf* cluster between them. Intriguingly, *X*ρ sequences are part of a clade with clusters that do not directly appear to be *Starship* associated. However, some species, such as *Aspergillus nomius* and *Aspergillus bombycis*, have what appear to be degraded captain sequences nearby, indicating that they likely represent derelict *Starships*. Furthermore, a fragmented cluster from *A. flavus* that is sister to *A. nomius* shows extended homology with the *A. nomius* cluster, indicating that this region is likely also *Starship* derived (Fig. 5A). Thus, we can infer that all *ssf* clusters have a single origin that coincides with their association to *Starships*, but that some clusters and cluster fragments have become integrated to the host background.

The *ssf* clusters in *M*ι and Πρ are very closely related and highly similar (>98% nucleotide identity for each *ssf* gene) despite the extremely distant relationships between their respective captains (~16% amino acid identity; Fig. 1). Furthermore, these two *Starships* share a considerable amount of common gene content beyond the formaldehyde resistance genes, including the *uvrA* gene (Fig. 5A) (*29*), and we previously observed that the *uvrA* gene is a putative DNA repair protein horizontally transferred into fungi from bacteria, which is restricted to a small number of fungal species (*29*). The notable similarity of cargo between two *Starships* from entirely different families strongly suggests that an inter-*Starship* recombination event has facilitated the de novo acquisition of the *ssf* cluster by one of these elements. Inferring the direction of HGT events is challenging because the phylogenies of captain and cargo genes often do not match the species tree at multiple internal nodes. The extended captain YR phylogeny presented in Fig. 1C, which incorporates captains from *Starships* that are closely related to those examined here (*40*), suggests that both *Starships* originated in *Aspergillus*, as they are most closely related to other *Aspergillus*-associated *Starships* and were subsequently transferred to *Paecilomyces, Penicillium*, or *Spiromastigoides*.

To support the assertion that the YR phylogeny can be used as evidence for the direction of horizontal transfer, we searched for additional clues by looking for cases where a smaller, species-specific TE may have jumped into the *Starship* before its transfer. Although Πρ is nearly identical between *Spiromastigoides* sp. and *P. variotii*, there is a unique insertion in *Spiromastigoides* sp. SCSIO F190 that corresponds to a nested LINE element (Fig. 1E). This element occurs only a single time in the genome of *Spiromastigoides* sp. SCSIO F190 but is multicopy within numerous strains of *A. sydowii* (table S4), suggesting that it has been associated with *A. sydowii* for a longer period of time. Furthermore, *Spiromastigoides* sp. SCSIO F190 has a second element belonging to the Prometheus-family that shows high similarity to *Starship *Πρ but lacks the *ssf* cluster (fig. S14). This *Starship* is nearly identical to one from *A. sydowii* CBS 593.65 (fig. S15). Lastly, *A. sydowii* MNP-2 also carries a copy of Πρ that is highly similar to the one form *A. cibarius* (except for a tandem duplication that includes the *ssf* cluster) (fig. S16). Together, this evidence strongly supports an origin of Πρ in *A. sydowii* or a close relative, with subsequent transfers to *Spiromastigoides* and *Paecilomyces*. As *A. sydowii* also has an *ssf*-carrying haplotype of *M*ι, this implies that Πρ acquired the cluster from *M*ι. In addition, the non-*Starship*–associated *ssf* cluster fragment in *A. nomius* and *A. bombycis* has some nucleotide similarity to the start of *M*ι (Fig. 5A), and so it may also be the case that *M*ι was the original donor of the *ssf* cluster to *Χ*ρ as well. As all *Starships* show evidence of HGT coincident with acquiring the *ssf* cluster, we infer that recurrent exchange of adaptive cargo genes between *Starships* promotes subsequent transfer to other lineages. The divergence of *T*υ relative to the other *ssf* clusters indicates that this process of repeated HGT has been occurring for extended amounts of evolutionary time, affecting the evolution of these fungi.

Among the *ssf* clusters identified (Fig. 5A), there is a spectrum of elements representing the stages in the “life cycle” of a *Starship* (Fig. 5C. Most straightforwardly, there are some *ssf* clusters nested within intact *Starships* such as the *X*ρ elements in *P. variotii* and *A. fumigatus*. In other cases, the *ssf* clusters are found within *Starships* that are mutated and no longer active. The *Spiromastix *Πρ in *P. variotii* IMV00236 and *Spiromastix sp*. SCSIOF190 are heavily affected by RIP mutation. We previously showed experimentally that if the starship *H*φ is duplicated within the genome, then it can be heavily mutated via RIP in *P. variotii* (*29*). The RIP mutation was not present within the *ssf* region, suggesting that the RIP occurred as a result of the presence of a second Πρ, lacking the ssf region, within the genome before the sexual cycle (fig. S14). Similarly, the *A. nomius* and *A. bombycis ssf* clusters are associated with a pseudogenised captain YR gene. Some other clusters show no *Starship* features yet are sister to *Starship*-associated sequences. We believe that these clusters—such as those in *A. flavus*, *A. pseudotamarii*, *Trichoderma*, *Fusarium*, and *Paecilomyces formosus*—represent former *Starship* cargo that has been integrated into the host genome. For example, we examined the cluster in *P. formosus*, which has no evidence of associated *Starship* sequence and is divergent from all other clusters. It is within this region that the *ssfA* homolog *fldA*, which was previously purified from *P. formosus* number 5, is found (*57*). The genomic region is an insertion relative to the sister species *P. maximus*. In each of the three available *P. formosus* genomes, the region shows different degrees of truncation and pseudogenization, suggesting that the region is actively being degraded. Notably, *ssfB* appears to be universally maintained, while other genes are pseudogenized or deleted (Fig. 5B). Given the observation that these genes have redundant functions with homologs in the genome of *P. variotii*, it suggests that selection is primarily maintaining genes, which uniquely contribute to formaldehyde resistance, tailoring the cluster to a specific fungal genomic landscape. Congruently, we see that the clade containing *A. flavus* sequences that are sister to *A. nomius* has only maintained *ssfA* and *ssfB*. Another piece of evidence that the *ssf* cluster in *P. formosus* is *Starship* derived is that genes *uvrA* and *ssfX* are present in the genome, although located elsewhere. The presence of *uvrA* is particularly compelling as this gene appears to have originally been acquired through an HGT event from bacteria and is only present in fungi that have the *ssf* gene cluster (being found in the *Starships* Πρ, *M*ι, and *T*υ) with the exception of *Phaeoacremonium* (fig. S17). The fact that horizontally and vertically inherited *Starship* cargo may eventually incorporate into the host genome is likely to lead to an underestimation of the importance of *Starships* in fungal evolution as these regions are difficult to recognize. Crucially, we have no evidence to suggest that the *ssf* cluster is an outlier when it comes to repeatedly associating with *Starships* because the dynamics uncovered here should be applicable to any fungal phenotype controlled by one or a few genes. Together with the vast array of functionality encoded by *Starship* cargo, this implies that the processes of gene acquisition and transfer by *Starships* are paramount to the evolution of fungi and their rapid adaptation to novel niches.

## DISCUSSION

### The *ssf* cluster supports the hypothesis that *Starship* HGT represent a powerful force in fungal adaptation

We now have a number of examples of HGT events from a wide diversity of eukaryotes including fungi (*14*–*17*, *20*, *22*, *23*, *30*, *58*, *59*). However, much about the role of HGT in eukaryotic biology remains unclear; for example, how HGT occurs and which parameters influence the frequency of observed HGT events (*60*). We do not know if there are active mechanisms of HGT in eukaryotes, similar to those in prokaryotes, which can mobilize genetic information between species at relatively high rates, repeatedly driving adaptation. Examining the *ssf* cluster demonstrates that *Starships* repeatedly facilitate the evolution of an adaptive trait through interelement cargo swapping and HGT, supporting a model in which both mobile element evolution and mobile element-mediated HGT play an important role in fungal evolution. We have found the *ssf* cluster to be present in numerous fungal species across different genera as a result of HGT (Fig. 1). The species in which the *ssf* cluster was found do not have known connections to a shared niche, and in some cases, they are known generalist species, for example, *A. fumigatus* and *P. variotii*. Moreover, we have found that at least four phylogenetically distant *Starships* have acquired and then mobilized the *ssf* cluster between fungal species following the swapping of cargo content between different *Starships*. It is remarkable to find a eukaryotic gene region so repeatedly associated with HGT with at least nine HGT events inferred. These transfers among four distinct *Starship* families essentially acts as four independent “replicates,” which confirm that acquisition of the formaldehyde resistance cluster primes the *Starships* for selection and maintenance following HGT between species. Given the relatively few publicly available genomes compared to extant fungal diversity, this figure likely massively understates the true number of HGTs of the *ssf* cluster driven by *Starships*.

### Other gene regions appear to follow the same evolutionary pattern as *ssf*

While the *ssf* gene cluster provides the most illustrative case study, two other examples of published *Starships* can now be interpreted to fit a similar evolutionary pattern (*30*, *31*).

The first is microbial host range driven by the *Starship Horizon *in pathogens of wheat (*31*). The *ToxA* gene encodes a proteinaceous host-specific toxin (TOXA), which enables pathogenicity on wheat cultivars with a particular susceptibility gene *Tsn1* (*61*). Since the mid-2000s, it has been clear that multiple wheat pathogens have utilized the common strategy of using the TOXA protein to infect susceptible wheat. These include *P. tritici-repentis* (*62*, *63*), *Parastagonospora nodorum* (*19*), and *Bipolaris sorokiniana* (*64*). The acquisition of *ToxA* by *P. tritici-repentis* likely occurred around 1940 and resulted in the emergence of this species as a new agent of disease in wheat (*19*). McDonald *et al.* (*65*) showed that in each of these species, *ToxA* was located within the 14-kb transposon ToxhAT, which, in each species, was internally embedded within a larger insertion relative to strains lacking *ToxA*. Because of the high level of sequence similarity within ToxhAT, HGT between these pathogens appeared to be the only reasonable hypothesis to explain its presence in multiple species (*19*). It was initially proposed that ToxhAT was responsible for mediating the HGT event (*31*, *65*). However, it has been recently found that the insertion within which ToxhAT is found is in fact a *Starship* named *Horizon* in both *P. tritici-repentis* and *Parastagonospora nodorum* and in an unrelated *Starship* named *Sanctuary* in *B. sorokiniana*, raising the alternative hypothesis of genetic exchanges between *Starships* follow by HGT (*31*, *66*). In light of the evolutionary dynamics and cargo swapping of *ssf*, which in the case of *ToxA* may be aided by the mobility of ToxhAT, we strongly favor the *Starship*-mediated hypothesis.

The second example of *Starship*-driven adaptation is the evolution of metal resistance in Eurotiales species through acquisition of Hephaestus-family *Starships*. Numerous Hephaestus-family *Starships* have independently acquired genes that encode resistance to various metals based on bioinformatic analysis of gene content. However, the best studied Hephaestus-family *Starship*, the type element *H*φ, found in *P. variotii*, confers resistance to at least five different metal ions—zinc, copper, arsenic, cadmium, and lead—mediated by four different genes, with cadmium and lead resistance as the result of a single transporter gene (*30*). As with *Starships* containing the *ssf* gene cluster, we found evidence for genetic exchanges and cargo swapping between *Starships*. Specifically, the genes required for arsenic resistance appear to have been introduced into *H*φ as the result of a nesting insertion event of another *Starship* (*30*). Initially, we identified a signal of HGT of this element between *P. variotii* and *Penicillium fuscoglucum* (*30*). Since then, we have observed additional HGT events between *P. variotii* and the *P. lecythidis* strain MCCF 102. This was a particularly notable example as the two copies of *H*φ differ by a single nucleotide across 86 kb (*29*). The sister species of *P. variotii* and *P. paravariotii* (only recently delimited from *P. variotii*) also contains apparent HGT-mediated acquisition of the element (*41*). Lastly, the examination of the recently released *P. chermesinum* IBT 19713 genome reveals yet another HGT of this element (fig. S4) (*50*). We thus have evidence for at least four HGT events of this *Starship* just in publicly available genomes and multiple examples of cargo exchanges between different elements.

In addition to these two examples which are explicitly described as *Starships*, there are already a number of HGT events described in the literature, which are likely to have been mediated by *Starships*. For example, we have noted that several of the HGT regions reported among cheese-making fungi are *Starships* (*28*, *29*). Similarly, a number of horizontally transferred regions in *Penicillium* fungi used to make salami were reported to contain the captain YR enzymes with the DUF3435 domain, which suggests that these regions of HGT are also *Starships* (*67*). The SORBUS gene cluster of *P. roqueforti* shows the presence/absence polymorphism among strains and increases resistance to sorbic acid. This region is suspected to have been introduced into the *P. roqueforti* genome via HGT and contains a DUF3723, which is a characteristic feature of *Starships* (*49*). The repeated association of *Starships* with gene clusters encoding the same or related metabolic pathways further suggests that the ability of these elements to horizontally transfer plays an important selective role in the formation and persistence of metabolic gene clusters in fungal genomes, as predicted by the selfish gene cluster and selfish operon hypotheses (*34*, *35*). Hence, the supposition that the *Starships* operate as active mechanisms of HGT and interact with each other to mediate recurrent, rapid adaptation and gene cluster formation beyond just formaldehyde resistance is a tangible avenue for future investigation.

### The selective advantage conferred by the cargo increases the probability of a *Starship* persisting following HGT

It is noteworthy that in each of the three examples of *Starship*-mediated HGT that have been best studied, namely, *ToxA*, metal resistance, and now formaldehyde resistance, HGT has occurred multiple times. The prevalence of HGT is particularly interesting, given that the majority of *Starship* evolution appears to follow a vertical pattern of inheritance (*28*). We hypothesize that *Starships* that gain host-beneficial cargo are primed for rapid dissemination through horizontal transfer events because these elements provide a competitive advantage to the recipient genome, and it is thus more likely that this recipient genome will pass its genes to the next generation and into the wider gene pool. In the absence of selective pressure, horizontally transferred elements are expected to be lost via genetic drift in most cases, same as with any other neutral or nearly neutral mutation. A feature shared by both the *ssf* gene cluster and *toxA* is that they are found within multiple distantly related *Starships*, suggesting that the reshuffling and swapping of adaptive cargo are common. Intermobile element gene shuffling is frequently observed in bacterial plasmids and integrative and conjugative elements and is a critical process, not only in generating new combinations of functional modules but also in promoting the maintenance and horizontal spread of ecologically and economically important phenotypes such as antibiotic resistance and nitrogen fixation (*68*–*70*). It remains to be seen how often *Starships* exchange cargo compared with their bacterial analogs, and what environments or genomic contexts might promote reshuffling.

### Formaldehyde is a ubiquitous organic compound found in a wide range of environments, making it difficult to understand in which environmental contexts the *ssf* cluster confers its adaptive advantage

The high level of identity between the *Starship* regions as shown in Fig. 1, beyond indicating the occurrence of HGT, implies that these events must have occurred in the recent past. It is thus interesting to consider the possible environmental factors driving the proliferation of this cluster. Formaldehyde is a ubiquitous organic compound and an important metabolite in biological systems [reviewed in (*71*)]. Formaldehyde is found in high concentration in some fruit and vegetables, which is one possible context in which saprotrophic members of the order Eurotiales might encounter formaldehyde (*72*). For example, it is produced by the activity of alcohol dehydrogenases on methanol (*73*). Formaldehyde is also an important anthropogenic pollutant, particularly of indoor air (*74*, *75*). Formaldehyde is toxic because it is highly reactive (*76*). Living organisms therefore have conserved mechanisms to limit this toxicity. In particular, the glutathione-dependent formaldehyde detoxification pathway is used by a wide range of organisms including bacteria, fungi, and other eukaryotes (*36*–*39*). Several studies have isolated formaldehyde-resistant fungal strains from formaldehyde-contaminated environments (*77*–*82*), suggesting that formaldehyde resistance might be rapidly evolving in response to environmental formaldehyde exposure. In a direct parallel to formaldehyde resistance mediated by *X*ρ, a conjugative plasmid has been demonstrated to transfer formaldehyde resistance between bacteria (*83*), suggesting that common selective pressures may act across both domains of life. However, given the wide range of sources of environmental formaldehyde, we cannot know in which environmental context the *ssf* cluster confers its selective advantage. Indeed, because formaldehyde is an intermediate in many biological pathways, the advantage of the *ssf* cluster may even be conferred as a result of metabolizing endogenously produced formaldehyde, rather than detoxification of external sources of formaldehyde. Recently, the *Starship Swordfish* linked environmental data to *Starship* presence/absence across over 1000 *Zymoseptoria* strains isolated from across the world and found an association between the *Starship* and various climatic variables (*84*). Future similar studies sequencing Eurotiales fungi directly isolated from different environmental contexts may be similarly illuminating in understanding the adaptive advantage of the *ssf* cluster in ecosystems.

### *Starship* evolution must be taken into account to understand fungal adaptation

Given that the acquisition of host beneficial gene cargo primes *Starships* for selection and maintenance following HGT events, the *Starships* hold a unique position in fungal evolutionary biology as agents of repeated adaptation via HGT. We show here that *Starship*-mediated HGT occurs repeatedly as opposed to in singular isolated events, indicating that HGT carried out by *Starships* is a powerful force in fungal adaptation and perhaps in the very formation and persistence of adaptive metabolic gene clusters, the implications of which we are just beginning to recognize. What we do not know is how *Starships* move between species, but hypothetically, it may involve the transfer of a circular intermediate (*29*, *66*).

As shown via examination of the *ssf* cluster, the expected “end point” in the life cycle of a *Starship* is that the cargo genes are integrated into the normal host genome, and identifying these regions in the absence of *Starship* features is difficult. For example, T-toxin virulence genes of *Cochliobolus heterostrophus* are located in a genomic region consistent with a degraded *Starship* but lacking conclusive evidence (*85*). Given the abundance of *Starships* in fungal genomes and their proclivity for horizontal transfer, we suggest that in all cases of fungal to fungal HGT, *Starship*-mediated transmission represents a plausible explanation. Giant transposable elements are found in other eukaryotes, for example, Teratorn elements are found in teleost fish, and *Maverick* elements are found in a wide range of eukaryotes from insects to ciliates (*86*, *87*). The HGT of a *Maverick* transposon has recently been reported in animals (*88*), and we suggest that similar processes may be observed in many eukaryotic phyla.

Evidence of HGT is widespread in fungi, yet the precise mechanisms of fungal horizontal transfer are poorly understood. Here, we have demonstrated that giant *Starship* transposons have facilitated the transfer of the *ssf* cluster between species at least nine different times, including between species belonging to different taxonomic orders. Drawing upon independent examples from four different *Starship* families, we show that *Starship*-mediated transfer of gene cargo between species is a recurrent event and fundamental property of *Starships* as opposed to an outlier event. While we show that *Starships* do transfer adaptive cargo, how they transfer remains largely unknown and will be an important future research direction. Given the diversity of threats and opportunities imparted by fungi and the potential for *Starships* to spread a wide range of gene cargos, they have implications on medicine, biosecurity, and conservation, which warrant urgent investigation.

## MATERIALS AND METHODS

### Strains

Five wild-type strains of *P. variotii* were examined, i.e., CBS 144490, CBS 101075 (*X*ρ^+^), FRR 1658, FRR 2889 (*X*ρ^+^), and FRR 3823, for formaldehyde resistance, all of which have available whole-genome sequencing data (*30*, *89*). Genetic manipulations were conducted in strain FRR 2889 because this strain contained a full-length *X*ρ region. *A. fumigatus* strains were selected from a recent pan genome study (*51*) including the only available *X*ρ^+^ strain C-1-80s-1 and 12 additional strains (all *X*ρ^−^) as controls, namely, C-1-76 s-1, L-2-15-4, B-1-70s-1, E-1-66 s-1, E-1-25-10, K-1-8 L-3, C-1-66 L-2, L-2-7-2, A-2-49 L-2, L-2-11-2, B-1-68 s-1, and K-1-48 s-1.

### *Paecilomyces* sexual crosses

*Paecilomyces* crossing was conducted as described previously (*89*, *90*). Strains of opposite mating type were plated on opposite sides of 90-mm petri dishes containing potato dextrose agar (PDA) and incubated in darkness at 30°C. Once ascospores were observed (up to 6 weeks), sexual spores were transferred to water and heat shocked at 80°C for 10 min to kill any contaminating conidia. The heat-treated spores were then plated onto CV8 agar and allowed to germinate for 2 days. The presence of *X*ρ within the progeny was assessed via PCR with duplex PCR using primer pair AUB662 + AUB663, which amplified 153 bp of the empty site of CBS 144490, and primer pair AUB651 + AUB653, which amplify 204 bp within *X*ρ.

### Formaldehyde sensitivity assay

Formaldehyde sensitivity was assessed using 10% cleared V8 agar pH 6 (CV8) for *P. variotii* and using PDA for *A. fumigatus* supplemented with formaldehyde in the form of formalin (37% formaldehyde in H_2_O, containing 10 to 15% methanol as stabilizer). Strains were grown on the formaldehyde-containing media for 3 days at 30°C before radial growth was assessed.

### Cloning

PCRs were conducted using Q5 High-Fidelity 2× Master according to the manufacturer’s directions, and the products were purified using the QIAquick Gel Extraction Kit. Specific details of the PCRs required for each construct are given in table S5. Templates were either plasmid DNA or genomic DNA extracted via a CTAB-based protocol from lyophilized mycelium (*91*).

Fragments for knockout/GFP replacement constructs were cloned into Eco RI/Hind III double-digested plasmid PLAUB36, which is the *Agrobacterium* binary vector pPZP-201BK modified to include the 2-μm origin and the *URA3* selectable marker for cloning in yeast (*29*). The cloning step was conducted using homologous recombination in yeast by transforming the digested vector and purified PCR products into strain BY4742 using a standard LiAc/PEG approach (*92*). Plasmids were rescued from the yeast and transferred to *Escherichia coli* using the Zymoprep Yeast Plasmid Miniprep Kit.

Complementation constructs were constructed by cloning the wildtype gene into the EcoRV site of plasmid pMAI2 (*93*) using the NEBuilder HiFi DNA Assembly Cloning Kit according to the manufacturer’s directions.

### Transformation

*Paecilomyces* was transformed as previously described (*89*). Briefly, plasmid constructs were introduced into *Agrobacterium* strain EHA105 by electroporation. *Paecilomyces* was then cocultured with the *Agrobacterium* on induction media containing acetosyringone before overlaying with selective media containing either hygromycin or G418 as appropriate for the construct. Transformant colonies were purified via single spore isolation before further analysis. In the case of gene knockouts, correct integration of the construct was assessed via PCR with the primers listed in table S6. The “upstream” primer was paired with primer AUB696 in GFP, or AUB113 in the promoter driving the G418 resistance gene, to produce a product unique to a correctly integrated construct. Similarly, the “downstream” primer was paired with AUB114 in the terminator of both the hygromycin and G418 resistance genes to produce a product unique to a correctly integrated construct.

### GFP expression reporter

Spores were germinated overnight in PDB with or without 0.1 μl/mL formalin in still culture at 30°C. Germlings were examined on microscope slides using a BioTek Cytation 1 imager. Settings were consistent across samples (4× objective GFP: LED intensity 5, integration time 350 ms, gain 0.6; Brightfield: LED intensity 5, integration time 38 ms, gain 0).

### Bioinformatic analysis

#### Blast-all inference of HGT

There are various methods by which HGT can be inferred. One approach is to use phylogenetic discordance, i.e., to look for genes whose phylogenetic history differs significantly from expected species histories (*94*). A weakness of phylogenies in the case of *Starships* is that these regions display the presence/absence polymorphisms so we cannot place these genes into the context of a set of vertically inherited homologs. However, we do see that both captain YR and *ssf* cargo trees are highly discordant with expected species relationships. Here, we have taken an alternative approach, which we refer to as BLAST-all. In this method, we take all the nucleotide gene sequences of one strain [predicted using Augustus implemented in Geneious Prime version 2023.0.4 with an *A. nidulans* gene model (*95*)] and use BLASTn to search these against the genomic scaffolds of another strain. We then take the top result for each gene and produce a scatter plot of identity versus alignment length. Genes that are more similar between two species will produce BLAST hits of greater length and higher identity. Genes that have recently transferred horizontally between species will be more similar than the rest of the genome, as they have diverged more recently compared with the rest of the genome. This phylogeny-agnostic approach of inferring HGT using sequence conservation in the context of whole-genome comparisons is well suited for detecting large chunks of recently transferred DNA fragments, similar to the approach taken to infer HGT of *Maverick* transposons between nematode species (*88*). An alternative explanation for the existence of conserved DNA between species is introgression, that is to say, hybridization between two species followed by repeated backcrossing until only the *Starship* remains. We do not think that this explanation is satisfying for at least two reasons. First, as we described previously, the *Pegasus Starship* contains duplicated DNA and has horizontally transferred into *P. variotii*, which is a species in which duplicated DNA is mutated during sexual crosses; thus, the required backcrossing cannot have taken place. Second, while hybridization events have been reported between different fungal species, to our knowledge, in all cases, these occur between species within the same genus (*96*–*98*). *Paecilomyces* and *Spiromastigoides* belong to separate orders. However, it remains possible that introgression plays a role in the transfer of the *ssf*-containing *Starships* between closely related species.

#### Sequence searches and phylogenetic tree building

We searched a database of 2857 publicly available genomes for homologs to *ssf* cluster genes using BLASTp (*e* value = 0.001) (table S1). Because of the large amount of initial hits recovered for each gene, we iteratively built phylogenetic trees and selected clades of interest for further analysis based on their proximity to focal *ssf* genes from *Starship*-associated clusters. Each clade of interest was aligned using the E-INS-I method implemented in MAFFT version 7.490, which is recommended for sequences where several conserved motifs are embedded in long unalignable regions (*99*) and automatically trimmed using ClipKIT (--mode kpic-smart-gap) (*100*). We built three maximum likelihood trees for each gene using IQ-TREE v2.0 with automated model selection, 1000 SH-aLRT tests and 1000 ultrafast rapid bootstraps (-m MFP -alrt 1000 -B 1000), and selected the one with the highest likelihood (*101*, *102*).

A similar approach was used to build phylogenies specifically for *ssf* cluster genes found in *Paecilomyces.* Genes homologous to those found in the *ssf* cluster were identified via BLASTn searches against the same set of publicly available *Paecilomyces* genomes examined by Urquhart and Idnurm (*41*). Each group of homologs was aligned using MAFFT version 7.490 (*99*), and then alignments were manually examined and trimmed to remove poorly aligned DNA. Alignments were then concatenated as follows. Those homologs clustered with other *ssf* genes were considered “core” homologs. The core homologs from each species were concatenated. The homologs found within a particular cluster were concatenated together. A tree was generated from the concatenated alignment using the default settings of IQ-TREE with branch supports calculated from 10000 ultrafast bootstraps (*101*, *102*). This phylogeny was compared to a species phylogeny taken directly from Urquhart and Idnurm (*41*).

#### Genomic neighborhood annotation

We searched for genomic neighborhoods containing clustered genes of interest using the complete set of BLASTp hits recovered for the *ssf* cluster genes from the 2899 genome data as input to the command “starfish sketch” part of the starfish workflow (*40*). Neighborhoods were defined as having hits to at least three different genes within 25,000-bp distance of each other. We extracted genes ±25,000 bp of these neighborhoods and included them as part of each neighborhood to better understand their genomic contexts (table S2).

#### Determination of the presence of Χρ in A. fumigatus and P. variotii genomes

Using the *A. fumigatus* PA1607 *X*ρ sequence as a reference, we mapped short read Illumina data from 512 previously sequenced *A. fumigatus* strains using the workflow “starfish coverage” and the aligner strobealign (*103*) to determine the presence/absence frequencies of this element across a large globally distributed collection of strains and found a total of one environmental strain from Germany (C-1-80s-1) and four clinical strains from Japan (12-7504462, IFM-59073, IFM-59777, and IFM-62516) with >90% read coverage across the reference sequence (*51*, *52*, *104*).

To confirm the presence or absence of *X*ρ in each strain in which formaldehyde resistance was tested, Illumina sequencing reads were mapped to the FRR 2889 *X*ρ using inbuilt read mapper of Geneious Prime using the “low sensitivity/fastest” setting. Read depth across the *X*ρ region was assessed for the presence/absence polymorphism.

#### Taxonomic determination of P. rudallense and A. sydowi

“*Pencillium* sp.” strain IBT 35674x (*50*) was identified as *A. rudallense* based on comparison of the *RPB2* sequence to those examined by Houbraken *et al.* (*48*). Similarly, “*Aspergillus* sp.” PB4102 was taxonomically identified as *A. sydowii* based on a phylogenetic comparison of the *RPB2* sequence to those examined by Jurjevic *et al.* (*43*). In both cases, the *RPB2* sequence of the strain of interest was extracted from the genome assembled using BLAST and then aligned to the *RPB2* sequences of a set of taxonomically defined strains using MAFFT version 7.490 (*99*). A tree was generated from the alignment using the default settings of IQ-TREE with branch supports calculated from 10000 ultrafast bootstraps (*101*, *102*).

## References

[1] R. E. Lenski, Experimental evolution and the dynamics of adaptation and genome evolution in microbial populations. ISME J. 11, 2181–2194 (2017). 28509909 10.1038/ismej.2017.69 PMC5607360

[2] L. M. Cook, I. J. Saccheri, The peppered moth and industrial melanism: Evolution of a natural selection case study. Heredity 110, 207–212 (2013). 23211788 10.1038/hdy.2012.92 PMC3668657

[3] F. C. Jones, M. G. Grabherr, Y. F. Chan, P. Russell, E. Mauceli, J. Johnson, R. Swofford, M. Pirun, M. C. Zody, S. White, E. Birney, S. Searle, J. Schmutz, J. Grimwood, M. C. Dickson, R. M. Myers, C. T. Miller, B. R. Summers, A. K. Knecht, S. D. Brady, H. Zhang, A. A. Pollen, T. Howes, C. Amemiya; Broad Institute Genome Sequencing Platform & Whole Genome Assembly Team, J. Baldwin, T. Bloom, D. B. Jaffe, R. Nicol, J. Wilkinson, E. S. Lander, F. Di Palma, K. Lindblad-Toh, D. M. Kingsley, The genomic basis of adaptive evolution in threespine sticklebacks. Nature 484, 55–61 (2012). 22481358 10.1038/nature10944 PMC3322419

[4] A. E. Henschen, M. Vinkler, M. M. Langager, A. A. Rowley, R. A. Dalloul, D. M. Hawley, J. S. Adelman, Rapid adaptation to a novel pathogen through disease tolerance in a wild songbird. PLOS Pathog. 19, e1011408 (2023). 37294834 10.1371/journal.ppat.1011408 PMC10287013

[5] C. L. Price, J. E. Parker, A. G. S. Warrilow, D. E. Kelly, S. L. Kelly, Azole fungicides—Understanding resistance mechanisms in agricultural fungal pathogens. Pest Manag. Sci. 71, 1054–1058 (2015). 25914201 10.1002/ps.4029

[6] G. M. Rossolini, F. Arena, P. Pecile, S. Pollini, Update on the antibiotic resistance crisis. Curr. Opin. Pharmacol. 18, 56–60 (2014). 25254623 10.1016/j.coph.2014.09.006

[7] N. J. White, Antimalarial drug resistance. J. Clin. Invest. 113, 1084–1092 (2004). 15085184 10.1172/JCI21682 PMC385418

[8] J. Hemingway, L. Field, J. Vontas, An overview of insecticide resistance. Science 298, 96–97 (2002). 12364782 10.1126/science.1078052

[9] R. D. H. Barrett, D. Schluter, Adaptation from standing genetic variation. Trends Ecol. Evol. 23, 38–44 (2008). 18006185 10.1016/j.tree.2007.09.008

[10] Y. Yang, S. J. Marcoft, L. M. Forsyth, J. Zhao, Z. Li, A. P. Van de Wouw, A. Idnurm, Sterol demethylation inhibitor fungicide resistance in *Leptosphaeria maculans* is caused by modifications in the regulatory region of *ERG11*. Plant Dis. 104, 1280–1290 (2020). 32202465 10.1094/PDIS-10-19-2088-RE

[11] M.-J. Xiang, J.-Y. Liu, P.-H. Ni, S. Wang, C. Shi, B. Wei, Y.-X. Ni, H.-L. Ge, *Erg11* mutations associated with azole resistance in clinical isolates of *Candida albicans*. FEMS Yeast Res. 13, 386–393 (2013). 23480635 10.1111/1567-1364.12042

[12] S. M. Soucy, J. Huang, J. P. Gogarten, Horizontal gene transfer: Building the web of life. Nat. Rev. Genet. 16, 472–482 (2015). 26184597 10.1038/nrg3962

[13] N. A. Lerminiaux, A. D. S. Cameron, Horizontal transfer of antibiotic resistance genes in clinical environments. Can. J. Microbiol. 65, 34–44 (2019). 30248271 10.1139/cjm-2018-0275

[14] J. Xia, Z. Guo, Z. Yang, H. Han, S. Wang, H. Xu, X. Yang, F. Yang, Q. Wu, W. Xie, X. Zhou, W. Dermauw, T. C. J. Turlings, Y. Zhang, Whitefly hijacks a plant detoxification gene that neutralizes plant toxins. Cell 184, 3588 (2021). 34171320 10.1016/j.cell.2021.06.010

[15] N. A. Moran, T. Jarvik, Lateral transfer of genes from fungi underlies carotenoid production in aphids. Science 328, 624–627 (2010). 20431015 10.1126/science.1187113

[16] L. A. Graham, J. Li, W. S. Davidson, P. L. Davies, Smelt was the likely beneficiary of an antifreeze gene laterally transferred between fishes. BMC Evol. Biol. 12, 190 (2012). 23009612 10.1186/1471-2148-12-190 PMC3499448

[17] L. A. Graham, P. L. Davies, Horizontal gene transfer in vertebrates: A fishy tale. Trends Genet. 37, 501–503 (2021). 33714557 10.1016/j.tig.2021.02.006

[18] U. L. Rosewich, H. C. Kistler, Role of horizontal gene transfer in the evolution of fungi. Annu. Rev. Phytopathol. 38, 325–363 (2000). 11701846 10.1146/annurev.phyto.38.1.325

[19] T. L. Friesen, E. H. Stukenbrock, Z. Liu, S. Meinhardt, H. Ling, J. D. Faris, J. B. Rasmussen, P. S. Solomon, B. A. McDonald, R. P. Oliver, Emergence of a new disease as a result of interspecific virulence gene transfer. Nat. Genet. 38, 953–956 (2006). 16832356 10.1038/ng1839

[20] K. Cheeseman, J. Ropars, P. Renault, J. Dupont, J. Gouzy, A. Branca, A.-L. Abraham, M. Ceppi, E. Conseiller, R. Debuchy, F. Malagnac, A. Goarin, P. Silar, S. Lacoste, E. Sallet, A. Bensimon, T. Giraud, Y. Brygoo, Multiple recent horizontal transfers of a large genomic region in cheese making fungi. Nat. Commun. 5, 2876 (2014). 24407037 10.1038/ncomms3876 PMC3896755

[21] J. Ropars, R. C. R. de la Vega, M. López-Villavicencio, J. Gouzy, E. Sallet, É. Dumas, S. Lacoste, R. Debuchy, J. Dupont, A. Branca, T. Giraud, Adaptive horizontal gene transfers between multiple cheese-associated fungi. Curr. Biol. 25, 2562–2569 (2015). 26412136 10.1016/j.cub.2015.08.025 PMC4598740

[22] G. J. Szöllősi, A. A. Davín, E. Tannier, V. Daubin, B. Boussau, Genome-scale phylogenetic analysis finds extensive gene transfer among fungi. Phil. Trans. R. Soc. B 370, 20140335 (2015). 26323765 10.1098/rstb.2014.0335 PMC4571573

[23] J. H. Wisecaver, J. C. Slot, A. Rokas, The evolution of fungal metabolic pathways. PLOS Genet. 10, e1004816 (2014). 25474404 10.1371/journal.pgen.1004816 PMC4256263

[24] J. H. Wisecaver, A. Rokas, Fungal metabolic gene clusters-caravans traveling across genomes and environments. Front. Microbiol. 6, 161 (2015). 25784900 10.3389/fmicb.2015.00161 PMC4347624

[25] T. A. Richards, D. M. Soanes, M. D. M. Jones, O. Vasieva, G. Leonard, K. Paszkiewicz, P. G. Foster, N. Hall, N. J. Talbot, Horizontal gene transfer facilitated the evolution of plant parasitic mechanisms in the oomycetes. Proc. Natl. Acad. Sci. U.S.A. 108, 15258–15263 (2011). 21878562 10.1073/pnas.1105100108 PMC3174590

[26] R. Dean, J. A. L. Van Kan, Z. A. Pretorius, K. E. Hammond-Kosack, A. Di Pietro, P. D. Spanu, J. J. Rudd, M. Dickman, R. Kahmann, J. Ellis, G. D. Foster, The top 10 fungal pathogens in molecular plant pathology. Mol. Plant Pathol. 13, 414–430 (2012). 22471698 10.1111/j.1364-3703.2011.00783.x PMC6638784

[27] K. Kainz, M. A. Bauer, F. Madeo, D. Carmona-Gutierrez, Fungal infections in humans: The silent crisis. Microb. Cell Fact. 7, 143–145 (2020). 10.15698/mic2020.06.718 PMC7278517 32548176

[28] E. Gluck-Thaler, T. Ralston, Z. Konkel, C. G. Ocampos, V. D. Ganeshan, A. E. Dorrance, T. L. Niblack, C. W. Wood, J. C. Slot, H. D. Lopez-Nicora, A. A. Vogan, Giant *Starship* elements mobilize accessory genes in fungal genomes. Mol. Biol. Evol. 39, msac109 (2022). 35588244 10.1093/molbev/msac109 PMC9156397

[29] A. S. Urquhart, A. A. Vogan, D. M. Gardiner, A. Idnurm, *Starships* are active eukaryotic transposable elements mobilized by a new family of tyrosine recombinases. Proc. Natl. Acad. Sci. U.S.A. 120, e2214521120 (2023). 37023132 10.1073/pnas.2214521120 PMC10104507

[30] A. S. Urquhart, N. F. Chong, Y. Yang, A. Idnurm, A large transposable element mediates metal resistance in the fungus *Paecilomyces variotii*. Curr. Biol. 32, 937–950.e5 (2022). 35063120 10.1016/j.cub.2021.12.048

[31] R. Gourlie, M. McDonald, M. Hafez, R. Ortega-Polo, K. E. Low, D. W. Abbott, S. E. Strelkov, F. Daayf, R. Aboukhaddour, The pangenome of the wheat pathogen *Pyrenophora tritici-repentis* reveals novel transposons associated with necrotrophic effectors *ToxA* and *ToxB*. BMC Biol. 20, 239 (2022). 36280878 10.1186/s12915-022-01433-w PMC9594970

[32] S. M. Tralamazza, E. Gluck-Thaler, A. Feurtey, D. Croll, Copy number variation introduced by a massive mobile element facilitates global thermal adaptation in a fungal wheat pathogen. Nat. Commun. 15, 5728 (2024). 38977688 10.1038/s41467-024-49913-7 PMC11231334

[33] E. Gluck-Thaler, A. A. Vogan, Systematic identification of cargo-mobilizing genetic elements reveals new dimensions of eukaryotic diversity. Nucleic Acids Res. 52, 5496–5513 (2024). 38686785 10.1093/nar/gkae327 PMC11162782

[34] J. D. Walton, Horizontal gene transfer and the evolution of secondary metabolite gene clusters in fungi: An hypothesis. Fungal Genet. Biol. 30, 167–171 (2000). 11035938 10.1006/fgbi.2000.1224

[35] J. G. Lawrence, J. R. Roth, Selfish operons: Horizontal transfer may drive the evolution of gene clusters. Genetics 143, 1843–1860 (1996). 8844169 10.1093/genetics/143.4.1843 PMC1207444

[36] W. G. Gutheil, B. Holmquist, B. L. Vallee, Purification, characterization, and partial sequence of the glutathione-dependent formaldehyde dehydrogenase from *Escherichia coli*: A class III alcohol dehydrogenase. Biochemistry 31, 475–481 (1992). 1731906 10.1021/bi00117a025

[37] P. Strittmatter, E. G. Ball, Formaldehyde dehydrogenase, a glutathione-dependent enzyme system. J. Biol. Chem. 213, 445–461 (1955). 14353946

[38] L. Uotila, M. Koivusalo, Purification of formaldehyde and formate dehydrogenases from pea seeds by affinity chromatography and *S*-formylglutathione as the intermediate of formaldehyde metabolism. Arch. Biochem. Biophys. 196, 33–45 (1979). 574372 10.1016/0003-9861(79)90548-4

[39] E. P. Wehner, E. Rao, M. Brendel, Molecular structure and genetic regulation of *SFA*, a gene responsible for resistance to formaldehyde in *Saccharomyces cerevisiae*, and characterization of its protein product. Mol. Gen. Genet. 237, 351–358 (1993). 8483449 10.1007/BF00279438

[40] E. Gluck-Thaler, A. A. Vogan, Systematic identification of cargo-carrying genetic elements reveals new dimensions of eukaryotic diversity. bioRxiv 563810 [Preprint] (2023). https://doi.org/10.1101/2023.10.24.563810. 10.1093/nar/gkae327 PMC11162782 38686785

[41] A. S. Urquhart, A. Idnurm, A polyphasic approach including whole genome sequencing reveals *Paecilomyces paravariotii* sp. nov. as a cryptic sister species to *P variotii*. J Fungi 9, 285 (2023). 10.3390/jof9030285 PMC10055108 36983453

[42] R. W. Bastos, C. Valero, L. P. Silva, T. Schoen, M. Drott, V. Brauer, R. Silva-Rocha, A. Lind, J. L. Steenwyk, A. Rokas, F. Rodrigues, A. Resendiz-Sharpe, K. Lagrou, M. Marcet-Houben, T. Gabaldón, E. McDonnell, I. Reid, A. Tsang, B. R. Oakley, F. V. Loures, F. Almeida, A. Huttenlocher, N. P. Keller, L. N. A. Ries, G. H. Goldman, Functional characterization of clinical isolates of the opportunistic fungal pathogen *Aspergillus nidulans*. mSphere 5, e0015320 (2020). 10.1128/mSphere.00153-20 PMC7142298 32269156

[43] Z. Jurjevic, S. W. Peterson, B. W. Horn, *Aspergillus* section *Versicolores*: Nine new species and multilocus DNA sequence based phylogeny. IMA Fungus 3, 59–79 (2012). 23155501 10.5598/imafungus.2012.03.01.07 PMC3399103

[44] H. Kandemir, K. Dukik, M. de Melo Teixeira, J. B. Stielow, F. Z. Delma, A. M. S. Al-Hatmi, S. A. Ahmed, M. Ilkit, G. S. de Hoog, Phylogenetic and ecological reevaluation of the order *Onygenales*. Fungal Divers. 115, 1–72 (2022).

[45] M. Shao, C. Sun, X. Liu, X. Wang, W. Li, X. Wei, Q. Li, J. Ju, Upregulation of a marine fungal biosynthetic gene cluster by an endobacterial symbiont. Commun. Biol. 3, 527 (2020). 32968175 10.1038/s42003-020-01239-y PMC7511336

[46] N. K. Singh, A. Blachowicz, J. Romsdahl, C. Wang, T. Torok, K. Venkateswaran, Draft genome sequences of several fungal strains selected for exposure to microgravity at the International Space Station. Genome Announc. 5, e0160216 (2017). 10.1128/genomeA.01602-16 PMC5391430 28408692

[47] E. Gladyshev, Repeat-induced point mutation and other genome defense mechanisms in fungi. Microbiol. Spectr. 5, 10.1128/microbiolspec.FUNK-0042-2017 (2017). PMC5607778 28721856

[48] J. Houbraken, C. M. Visagie, M. Meijer, J. C. Frisvad, P. E. Busby, J. I. Pitt, K. A. Seifert, G. Louis-Seize, R. Demirel, N. Yilmaz, K. Jacobs, M. Christensen, R. A. Samson, A taxonomic and phylogenetic revision of *Penicillium* section *Aspergilloides*. Stud. Mycol. 78, 373–451 (2014). 25492984 10.1016/j.simyco.2014.09.002 PMC4255628

[49] M. Punt, S. J. Seekles, J. L. van Dam, C. de Adelhart Toorop, R. R. Martina, J. Houbraken, A. F. J. Ram, H. A. B. Wösten, R. A. Ohm, High sorbic acid resistance of *Penicillium roqueforti* is mediated by the SORBUS gene cluster. PLOS Genet. 18, e1010086 (2022). 35704633 10.1371/journal.pgen.1010086 PMC9200314

[50] C. Petersen, T. Sørensen, M. R. Nielsen, T. E. Sondergaard, J. L. Sørensen, D. A. Fitzpatrick, J. C. Frisvad, K. L. Nielsen, Comparative genomic study of the *Penicillium* genus elucidates a diverse pangenome and 15 lateral gene transfer events. IMA Fungus 14, 3 (2023). 36726175 10.1186/s43008-023-00108-7 PMC9893605

[51] A. E. Barber, T. Sae-Ong, K. Kang, B. Seelbinder, J. Li, G. Walther, G. Panagiotou, O. Kurzai, *Aspergillus fumigatus* pan-genome analysis identifies genetic variants associated with human infection. Nat. Microbiol. 6, 1526–1536 (2021). 34819642 10.1038/s41564-021-00993-x

[52] L. A. Lofgren, B. S. Ross, R. A. Cramer, J. E. Stajich, The pan-genome of *Aspergillus fumigatus* provides a high-resolution view of its population structure revealing high levels of lineage-specific diversity driven by recombination. PLOS Biol. 20, e3001890 (2022). 36395320 10.1371/journal.pbio.3001890 PMC9714929

[53] C. Umansky, A. E. Morellato, M. Rieckher, M. A. Scheidegger, M. R. Martinefski, G. A. Fernández, O. Pak, K. Kolesnikova, H. Reingruber, M. Bollini, G. P. Crossan, N. Sommer, M. E. Monge, B. Schumacher, L. B. Pontel, Endogenous formaldehyde scavenges cellular glutathione resulting in redox disruption and cytotoxicity. Nat. Commun. 13, 745 (2022). 35136057 10.1038/s41467-022-28242-7 PMC8827065

[54] N. Couto, J. Wood, J. Barber, The role of glutathione reductase and related enzymes on cellular redox homoeostasis network. Free Radic. Biol. Med. 95, 27–42 (2016). 26923386 10.1016/j.freeradbiomed.2016.02.028

[55] M. Goenrich, S. Bartoschek, C. H. Hagemeier, C. Griesinger, J. A. Vorholt, A glutathione-dependent formaldehyde-activating enzyme (Gfa) from *Paracoccus denitrificans* detected and purified via two-dimensional proton exchange NMR spectroscopy. J. Biol. Chem. 277, 3069–3072 (2002). 11741920 10.1074/jbc.C100579200

[56] R. J. Hopkinson, I. K. H. Leung, T. J. Smart, N. R. Rose, L. Henry, T. D. W. Claridge, C. J. Schofield, Studies on the glutathione-dependent formaldehyde-activating enzyme from *Paracoccus denitrificans*. PLOS ONE 10, e0145085 (2015). 26675168 10.1371/journal.pone.0145085 PMC4682968

[57] T. Oka, Y. Komachi, K. Ohshima, Y. Kawano, K. Fukuda, K. Nagahama, K. Ekino, Y. Nomura, Isolation, sequencing, and heterologous expression of the *Paecilomyces variotii* gene encoding *S*-hydroxymethylglutathione dehydrogenase (*fldA*). Appl. Microbiol. Biotechnol. 99, 1755–1763 (2015). 25398285 10.1007/s00253-014-6203-8 PMC4322224

[58] H. Qiu, G. Cai, J. Luo, D. Bhattacharya, N. Zhang, Extensive horizontal gene transfers between plant pathogenic fungi. BMC Biol. 14, 41 (2016). 27215567 10.1186/s12915-016-0264-3 PMC4876562

[59] D. A. Fitzpatrick, Horizontal gene transfer in fungi. FEMS Microbiol. Lett. 329, 1–8 (2012). 22112233 10.1111/j.1574-6968.2011.02465.x

[60] P. J. Keeling, Horizontal gene transfer in eukaryotes: Aligning theory with data. Nat. Rev. Genet. 25, 416–430 (2024). 38263430 10.1038/s41576-023-00688-5

[61] L. M. Ciuffetti, R. P. Tuori, J. M. Gaventa, A single gene encodes a selective toxin causal to the development of tan spot of wheat. Plant Cell 9, 135–144 (1997). 9061946 10.1105/tpc.9.2.135 PMC156906

[62] A. Tomas, G. H. Feng, G. R. Reeck, W. W. Bockus, J. E. Leach, Purification of a cultivar-specific toxin from *Pyrenophora tritici-repentis*, causal agent of tan spot of wheat. Mol. Plant Microbe Interact. 3, 221–224 (1990).

[63] G. M. Ballance, L. Lamari, C. C. Bernier, Purification and characterization of a host-selective necrosis toxin from *Pyrenophora tritici-repentis*. Physiol. Mol. Plant Pathol. 35, 203–213 (1989).

[64] M. C. McDonald, D. Ahren, S. Simpfendorfer, A. Milgate, P. S. Solomon, The discovery of the virulence gene *ToxA* in the wheat and barley pathogen *Bipolaris sorokiniana*. Mol. Plant Pathol. 19, 432–439 (2018). 28093843 10.1111/mpp.12535 PMC6638140

[65] M. C. McDonald, A. P. Taranto, E. Hill, B. Schwessinger, Z. Liu, S. Simpfendorfer, A. Milgate, P. S. Solomon, Transposon-mediated horizontal transfer of the host-specific virulence protein ToxA between three fungal wheat pathogens. MBio 10, e0151519 (2019). 10.1128/mBio.01515-19 PMC6737239 31506307

[66] A. H. Bucknell, M. C. McDonald, That’s no moon, it’s a *Starship*: Giant transposons driving fungal horizontal gene transfer. Mol. Microbiol. 120, 555–563 (2023). 37434470 10.1111/mmi.15118

[67] Y.-C. Lo, J. Bruxaux, R. C. R. de la Vega, S. O’Donnell, A. Snirc, M. Coton, M. Le Piver, S. Le Prieur, D. Roueyre, J. Dupont, J. Houbraken, R. Debuchy, J. Ropars, T. Giraud, A. Branca, Domestication in dry-cured meat *Penicillium* fungi: Convergent specific phenotypes and horizontal gene transfers without strong genetic subdivision. Evol. Appl. 16, 1637–1660 (2023). 37752962 10.1111/eva.13591 PMC10519415

[68] A. J. Weisberg, A. Rahman, D. Backus, P. Tyavanagimatt, J. H. Chang, J. L. Sachs, Pangenome evolution reconciles robustness and instability of rhizobial symbiosis. mBio 13, e0007422 (2022). 35416699 10.1128/mbio.00074-22 PMC9239051

[69] M. W. Pesesky, R. Tilley, D. A. C. Beck, Mosaic plasmids are abundant and unevenly distributed across prokaryotic taxa. Plasmid 102, 10–18 (2019). 30797764 10.1016/j.plasmid.2019.02.003

[70] J. T. Shapiro, A. Zorea, A. B. Kav, V. J. Ontiveros, I. Mizrahi, S. Pilosof, Multilayer networks of plasmid genetic similarity reveal potential pathways of gene transmission. ISME J. 17, 649–659 (2023). 36759552 10.1038/s41396-023-01373-5 PMC10119158

[71] H. Reingruber, L. B. Pontel, Formaldehyde metabolism and its impact on human health. Curr. Opin. Toxicol. 9, 28–34 (2018).

[72] F. Nowshad, M. N. Islam, M. S. Khan, Concentration and formation behavior of naturally occurring formaldehyde in foods. Agric. Food Secur. 7, 17 (2018).

[73] W. Zhang, T. Zhang, S. Wu, M. Wu, F. Xin, W. Dong, J. Ma, M. Zhang, M. Jiang, Guidance for engineering of synthetic methylotrophy based on methanol metabolism in methylotrophy. RSC Adv. 7, 4083–4091 (2017).

[74] K.-H. Kim, S. A. Jahan, J.-T. Lee, Exposure to formaldehyde and its potential human health hazards. J. Environ. Sci. Health C 29, 277–299 (2011). 10.1080/10590501.2011.629972 22107164

[75] X. Tang, Y. Bai, A. Duong, M. T. Smith, L. Li, L. Zhang, Formaldehyde in China: Production, consumption, exposure levels, and health effects. Environ. Int. 35, 1210–1224 (2009). 19589601 10.1016/j.envint.2009.06.002

[76] N. H. Chen, K. Y. Djoko, F. J. Veyrier, A. G. McEwan, Formaldehyde stress responses in bacterial pathogens. Front. Microbiol. 7, 257 (2016). 26973631 10.3389/fmicb.2016.00257 PMC4776306

[77] T. Oka, K. Ekino, K. Fukuda, Y. Nomura, Draft genome sequence of the formaldehyde-resistant fungus *Byssochlamys spectabilis* No. 5 (anamorph *Paecilomyces variotii* No. 5) (NBRC109023). Genome Announc. 2, e0116213 (2014). 10.1128/genomeA.01162-13 PMC3886963 24407650

[78] H. Anton, H. Al-Ghoshae, M. Changmai, M. F. Baobaid, T. Aung, M. Al-Hoot, A. Al-Kabsi, Formalin resistant fungi isolated from cadavers at a medical school’s dissection hall in Malaysia. Asian J. Med. Health Sci. 5, 55–62 (2022).

[79] S. Kizil, A. U. Onel, E. M. Cecen, Isolation of fungus from the cadaver storage pool. Int. J. Agric. Res. 5, 85–91 (2022).

[80] D. S. Yu, G. Song, L. L. Song, W. Wang, C. H. Guo, Formaldehyde degradation by a newly isolated fungus *Aspergillus* sp. HUA. Int. J. Environ. Sci. Technol. 12, 247–254 (2015).

[81] D. Yu, L. Song, W. Wang, C. Guo, Isolation and characterization of formaldehyde-degrading fungi and its formaldehyde metabolism. Environ. Sci. Pollut. Res. Int. 21, 6016–6024 (2014). 24464080 10.1007/s11356-014-2543-2

[82] K. Sakaguchi, T. Inoue, S. Tada, On the production of aethyleneoxide-α, β-dicarboxylic acid by moulds. Zentralbl. Bakteriol. Orig. 100, 302–307 (1939).

[83] P.-M. Kaulfers, D. Brandt, Isolation of a conjugative plasmid in *Escherichia coli* determining formaldehyde resistance. FEMS Microbiol. Lett. 43, 161–163 (1987).

[84] S. M. Tralamazza, E. Gluck-Thaler, A. Feurtey, D. Croll, Copy number variation introduced by a massive mobile element underpins global thermal adaptation in a fungal wheat pathogen. bioRxiv 559077 [Preprint] (2023). 10.1101/2023.09.22.559077. PMC11231334 38977688

[85] S. Haridas, J. B. González, R. Riley, M. Koriabine, M. Yan, V. Ng, A. Rightmyer, I. V. Grigoriev, S. E. Baker, B. G. Turgeon, T-Toxin virulence genes: Unconnected dots in a sea of repeats. MBio 14, e0026123 (2023). 36883814 10.1128/mbio.00261-23 PMC10128009

[86] Y. Inoue, M. Kumagai, X. Zhang, T. Saga, D. Wang, A. Koga, H. Takeda, Fusion of *piggyBac*-like transposons and herpesviruses occurs frequently in teleosts. Zool. Lett. 4, 6 (2018). 10.1186/s40851-018-0089-8 PMC5822658 29484202

[87] E. J. Pritham, T. Putliwala, C. Feschotte, *Mavericks*, a novel class of giant transposable elements widespread in eukaryotes and related to DNA viruses. Gene 390, 3–17 (2007). 17034960 10.1016/j.gene.2006.08.008

[88] S. A. Widen, I. C. Bes, A. Koreshova, P. Pliota, D. Krogull, A. Burga, Virus-like transposons cross the species barrier and drive the evolution of genetic incompatibilities. Science 380, eade0705 (2023). 37384706 10.1126/science.ade0705

[89] A. S. Urquhart, S. J. Mondo, M. R. Mäkelä, J. K. Hane, A. Wiebenga, G. He, S. Mihaltcheva, J. Pangilinan, A. Lipzen, K. Barry, R. P. de Vries, I. V. Grigoriev, A. Idnurm, Genomic and genetic insights into a cosmopolitan fungus, *Paecilomyces variotii* (Eurotiales). Front. Microbiol. 9, 3058 (2018). 30619145 10.3389/fmicb.2018.03058 PMC6300479

[90] J. Houbraken, J. Varga, E. Rico-Munoz, S. Johnson, R. A. Samson, Sexual reproduction as the cause of heat resistance in the food spoilage fungus *Byssochlamys spectabilis* (anamorph *Paecilomyces variotii*). Appl. Environ. Microbiol. 74, 1613–1619 (2008). 18192427 10.1128/AEM.01761-07 PMC2258620

[91] J. W. Pitkin, D. G. Panaccione, J. D. Walton, A putative cyclic peptide efflux pump encoded by the *TOXA* gene of the plant-pathogenic fungus *Cochliobolus carbonum*. Microbiology 142, 1557–1565 (1996). 8704997 10.1099/13500872-142-6-1557

[92] T. M. Joska, A. Mashruwala, J. M. Boyd, W. J. Belden, A universal cloning method based on yeast homologous recombination that is simple, efficient, and versatile. J. Microbiol. Methods 100, 46–51 (2014). 24418681 10.1016/j.mimet.2013.11.013 PMC4521215

[93] A. Idnurm, A. S. Urquhart, D. R. Vummadi, S. Chang, A. P. Van de Wouw, F. J. López-Ruiz, Spontaneous and CRISPR/Cas9-induced mutation of the osmosensor histidine kinase of the canola pathogen *Leptosphaeria maculans*. Fungal Biol. Biotechnol. 4, 12 (2017). 29270298 10.1186/s40694-017-0043-0 PMC5732519

[94] M. Ravenhall, N. Škunca, F. Lassalle, C. Dessimoz, Inferring horizontal gene transfer. PLOS Comput. Biol. 11, e1004095 (2015). 26020646 10.1371/journal.pcbi.1004095 PMC4462595

[95] M. Stanke, S. Waack, Gene prediction with a hidden Markov model and a new intron submodel. Bioinformatics 19, ii215 (2003). 14534192 10.1093/bioinformatics/btg1080

[96] D. P. Bendixsen, J. G. Frazão, R. Stelkens, *Saccharomyces* yeast hybrids on the rise. Yeast 39, 40–54 (2022). 34907582 10.1002/yea.3684

[97] M. Bovers, F. Hagen, E. E. Kuramae, M. R. Diaz, L. Spanjaard, F. Dromer, H. L. Hoogveld, T. Boekhout, Unique hybrids between the fungal pathogens *Cryptococcus neoformans* and *Cryptococcus gattii*. FEMS Yeast Res. 6, 599–607 (2006). 16696655 10.1111/j.1567-1364.2006.00082.x

[98] J. Steensels, B. Gallone, K. J. Verstrepen, Interspecific hybridization as a driver of fungal evolution and adaptation. Nat. Rev. Microbiol. 19, 485–500 (2021). 33767366 10.1038/s41579-021-00537-4

[99] K. Katoh, D. M. Standley, MAFFT multiple sequence alignment software version 7: Improvements in performance and usability. Mol. Biol. Evol. 30, 772–780 (2013). 23329690 10.1093/molbev/mst010 PMC3603318

[100] J. L. Steenwyk, T. J. Buida III, Y. Li, X.-X. Shen, A. Rokas, ClipKIT: A multiple sequence alignment trimming software for accurate phylogenomic inference. PLOS Biol. 18, e3001007 (2020). 33264284 10.1371/journal.pbio.3001007 PMC7735675

[101] B. Q. Minh, H. A. Schmidt, O. Chernomor, D. Schrempf, M. D. Woodhams, A. von Haeseler, R. Lanfear, IQ-TREE 2: New models and efficient methods for phylogenetic inference in the genomic era. Mol. Biol. Evol. 37, 1530–1534 (2020). 32011700 10.1093/molbev/msaa015 PMC7182206

[102] D. T. Hoang, O. Chernomor, A. von Haeseler, B. Q. Minh, L. S. Vinh, UFBoot2: Improving the ultrafast bootstrap approximation. Mol. Biol. Evol. 35, 518–522 (2018). 29077904 10.1093/molbev/msx281 PMC5850222

[103] K. Sahlin, Strobealign: Flexible seed size enables ultra-fast and accurate read alignment. Genome Biol. 23, 260 (2022). 36522758 10.1186/s13059-022-02831-7 PMC9753264

[104] E. Gluck-Thaler, A. Forsythe, C. Puerner, J. E. Stajich, D. Croll, R. A. Cramer, A. A. Vogan, Giant transposons promote strain heterogeneity in a major fungal pathogen. bioRxiv 601215 [Preprint] (2024). https://doi.org/10.1101/2024.06.28.601215. 10.1128/mbio.01092-25 PMC12153272 40353686

[105] A. C. E. Darling, B. Mau, F. R. Blattner, N. T. Perna, Mauve: Multiple alignment of conserved genomic sequence with rearrangements. Genome Res. 14, 1394–1403 (2004). 15231754 10.1101/gr.2289704 PMC442156

[106] S. Henikoff, J. G. Henikoff, Amino acid substitution matrices from protein blocks. Proc. Natl. Acad. Sci. U.S.A. 89, 10915–10919 (1992). 1438297 10.1073/pnas.89.22.10915 PMC50453

[107] L. Uotila, M. Koivusalo, Formaldehyde dehydrogenase from human liver: Purification, properties, and evidence for the formation of glutathione thiol esters by the enzyme. J. Biol. Chem. 249, 7653–7663 (1974). 4373474

[108] M. Ando, T. Yoshimoto, S. Ogushi, K. Rikitake, S. Shibata, D. Tsuru, Formaldehyde dehydrogenase from *Pseudomonas putida*. Purification and some properties. J. Biochem. 85, 1165–1172 (1979). 571868

[109] K. Ito, M. Takahashi, T. Yoshimoto, D. Tsuru, Cloning and high-level expression of the glutathione-independent formaldehyde dehydrogenase gene from *Pseudomonas putida*. J. Bacteriol. 176, 2483–2491 (1994). 8169197 10.1128/jb.176.9.2483-2491.1994 PMC205383

[110] A. Minemura, R. Kitamura, M. Sano, S. Osawa, The gene of *Aspergillus oryzae* involved in degradation of formaldehyde and formaldehyde degradation in vapour phase by porous enzyme/chitosan nanofibre composites. J. Polym. Environ. 25, 1273–1279 (2017).

