# Supplementary material for: Gene acquisition by giant transposons primes eukaryotes for rapid evolution via horizontal gene transfer
Source: Sci Adv. 2024 Dec 6;10(49):eadp8738. doi: 10.1126/sciadv.adp8738 (PMC11623301; doi:10.1126/sciadv.adp8738)
Supplement: Supplementary file 1 — Figs. S1 to S17 Tables S1 to S3 and S6 to S8 Legends for tables S4 and S5 [file sciadv.adp8738_sm.pdf]

Science Advances
AAAS

Supplementary Materials for

Gene acquisition by giant transposons primes eukaryotes for rapid evolution via horizontal gene transfer

Andrew S. Urquhart *et al.*

Corresponding author: Andrew S. Urquhart, andrew.urquhart@ebc.uu.se; Aaron A. Vogan, aaron.vogan@ebc.uu.se

Sci. Adv. **10**, eadp8738 (2024)
DOI: 10.1126/sciadv.adp8738

The PDF file includes:

Figs. S1 to S17
Tables S1 to S3 and S6 to S8
Legends for tables S4 and S5

Other Supplementary Material for this manuscript includes the following:

Tables S4 and S5

Supplementary Figures

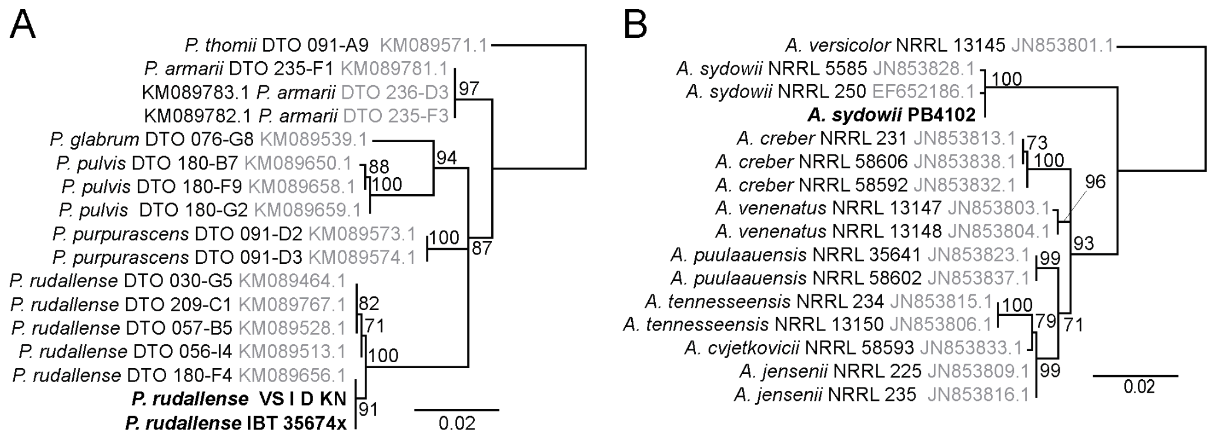

Figure S1: Taxonomic determination of *P. rudallense* and *A. sydowii*. A) Phylogenetic tree based on RPB2 nucleotide sequence including *P. rudallense* and closely related *Penicillium* species. B) Phylogenetic tree based on RPB2 nucleotide sequence including *P. sydowii* and closely related *Aspergillus* species. Both trees generated in IQ-TREE. Ultrafast bootstraps from 1000 replicates indicated.

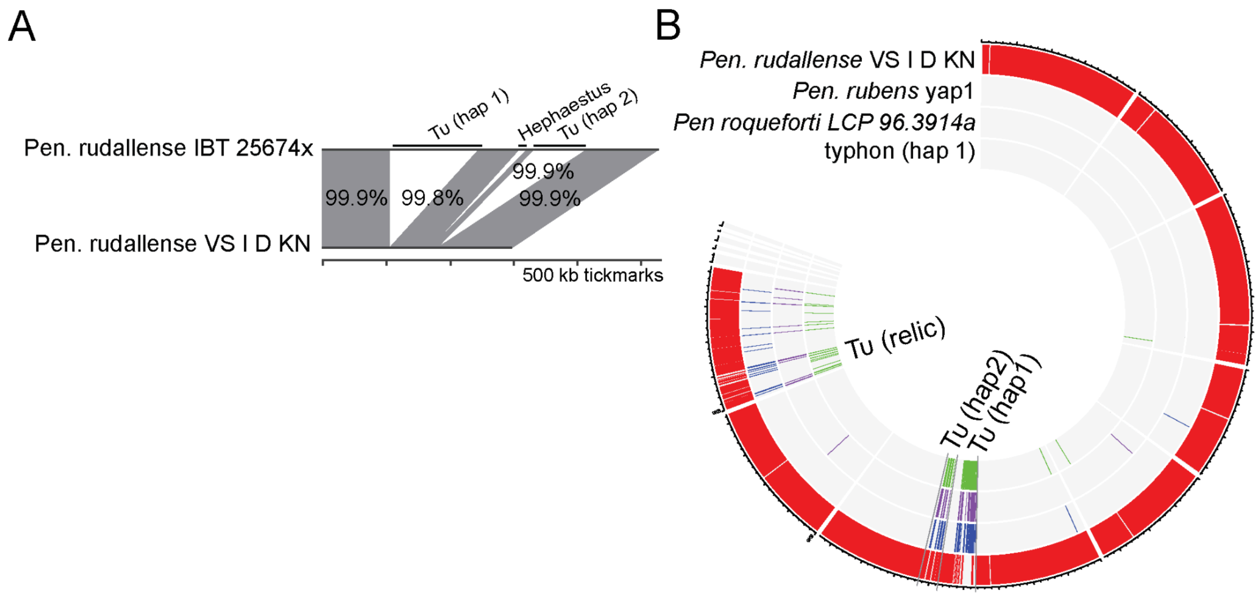

Figure S2: *Penicillium rudallense* strain IBT 25674x contains two *Typhon* (*Tu*) haplotypes *in* close proximity which evidence of horizontal transfer. A) Alignment of the *P. rudallense* IBT 35674x contig containing *Tu* and a contig from *P. rudallense* VS I D KN containing the corresponding empty site. Three large insertions are present in *P. rudallense* IBT 35674x. Two of these are *Tu* Starships and one is an Hephaestus-family *Starship*. **B)** Distribution of *P. rudallense* IBT 35674x genes returning BLAST hits with length >100 bp and identity >97% from databases consisting of 3 genomes the conspecific strain VS I D KN. *P. rubens* YAP1, *P. roqueforti* strain LCP 96.3914a, or a database consisting of only the *Tu* haplotype 1 sequence.

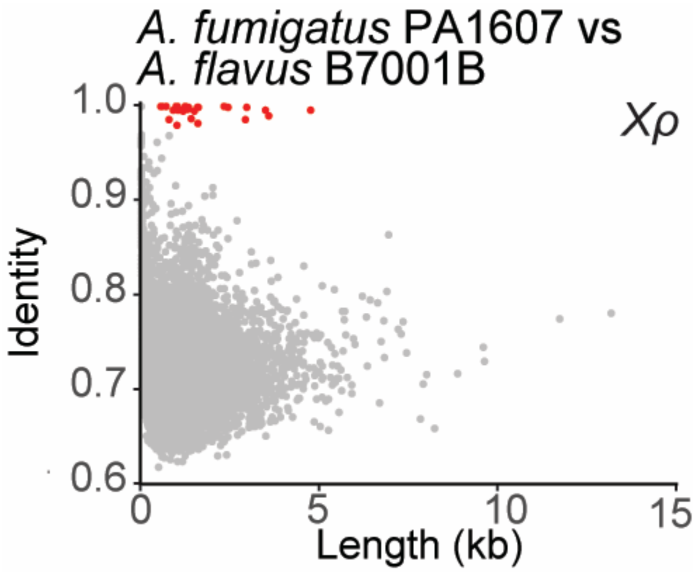

Figure S3: The *Chrysaor* (*Xp*) *Starship* of *A. fumigatus* PA1607 and *A. flavus* B7001B show highly sequence similarity consistent with HGT. Shown is a BLAST-all comparison of *A. fumigatus* PA1607 and *A. flavus* B7001B as per Figure 1D, with the red dots being those found within *Xp*.

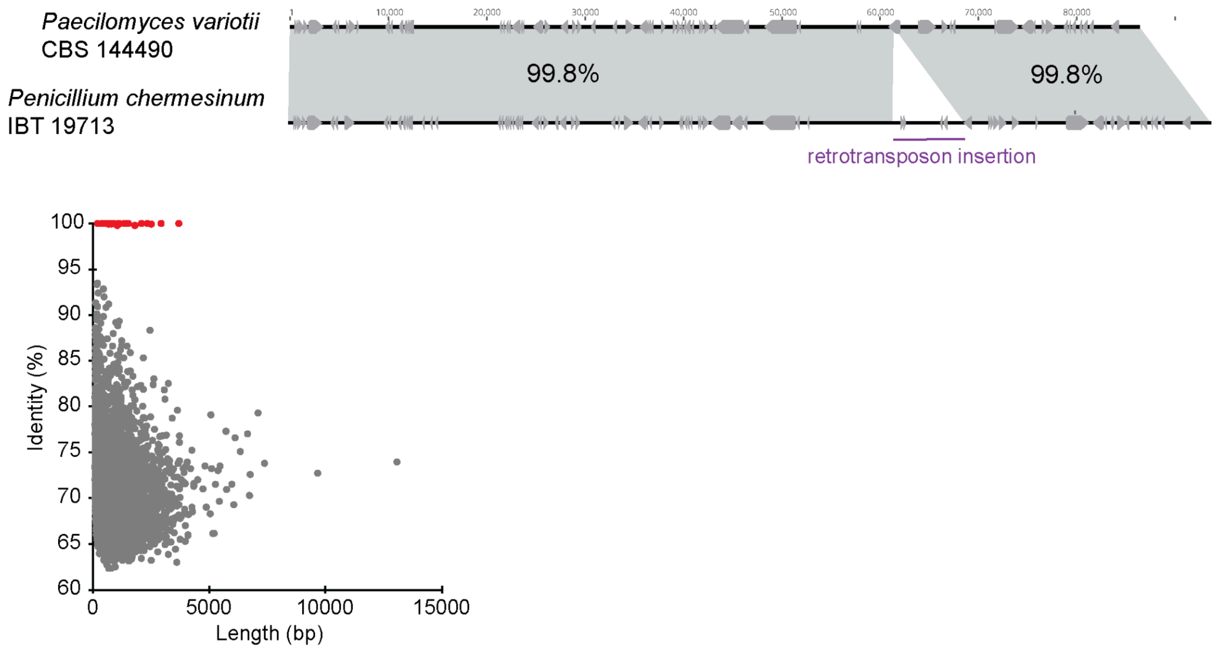

Figure S4: *Paecilomyces variotii* CBS 144490 and *Penicillium chermesinum* IBT 19713 contain near identical copies of the metal resistance *Hephaestus* (*Hφ*) *Starship* except for the insertion of a retrotransposon into *P. chermesinum*. The top panel shows the nucleotide alignment between the two copies and bottom panel the outcome of BLAST of all gene sequences, with the red dots being those found within *Hφ*.

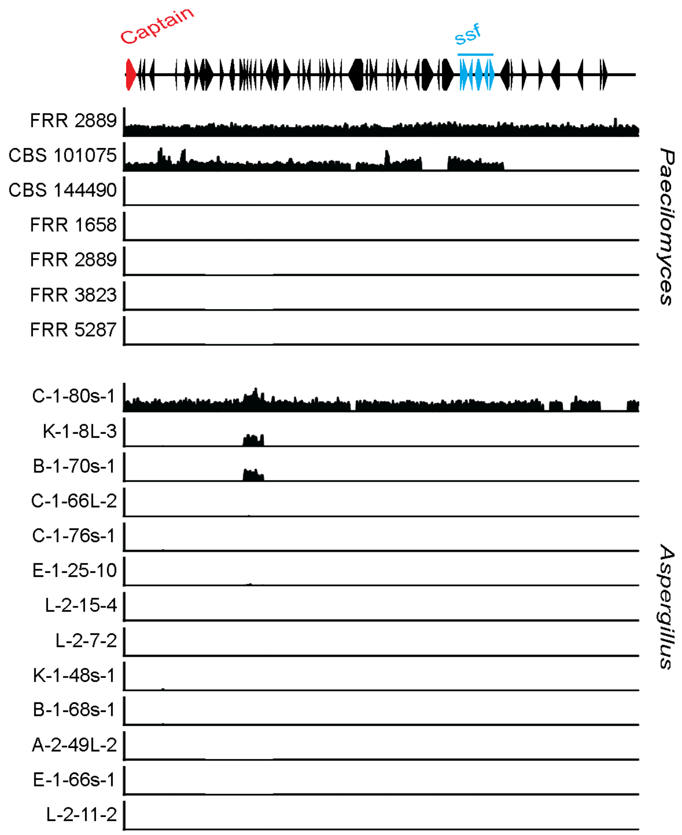

Figure S5: Illumina read mapping demonstrated the presence or absence of Chrysaor *Xp* within a set of *Paecillomyces variotii* and *Aspergillus fumigatus* strains. Shown is Illumina reads (5 million for each strain) mapped to the FRR 2889 *Xp* sequence. Y-axis represents read depth between 0 and 100× coverage.

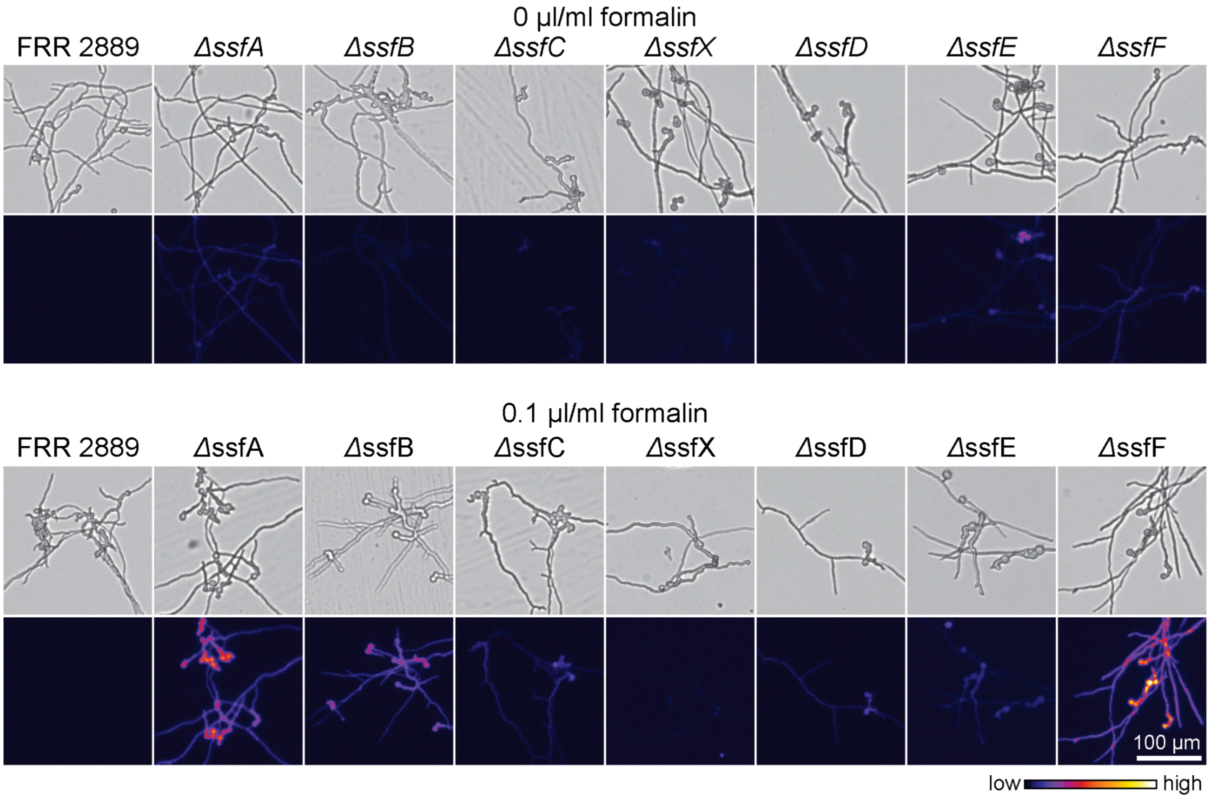

Figure S6: The expression of some *ssf* genes is strongly induced by formaldehyde. GFP fluorescence in wild type and GFP replacement mutants germinated overnight in potato dextrose broth with and without supplementation with 0.1 µl/ml formalin.

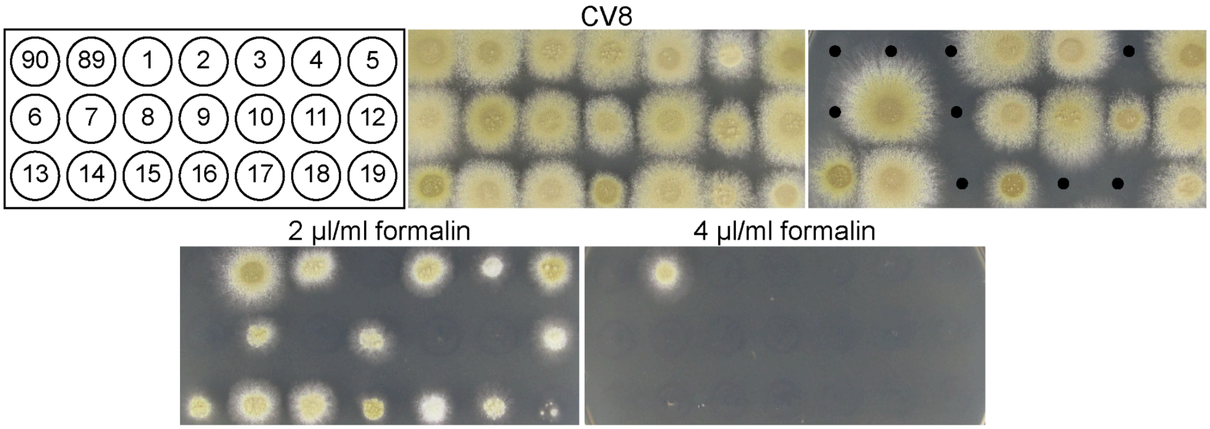

Figure S7: A second source of formaldehyde resistance segregates independently from *Chrysaor Xρ*. 19 progeny of a cross between CBS 144490 and FRR 2889 *ΔssfA-F* grown on cleared V8 agar (CV8), media supplemented with hygromycin (resistance is linked to *Xρ*), and media supplemented with 2 µl/ml or 4 µl/ml formalin. No progeny grow on 4 µl/ml formalin and on 2 µl/ml formalin resistance segregates independently to *Xρ*/hygromycin resistance. Black dots added to indicate lack of growth on hygromycin. The wildtype strains CBS 144490 (“90”) and FRR 2889 (“89”) are shown for comparison. Plates were incubated for 3 days at 30°C.

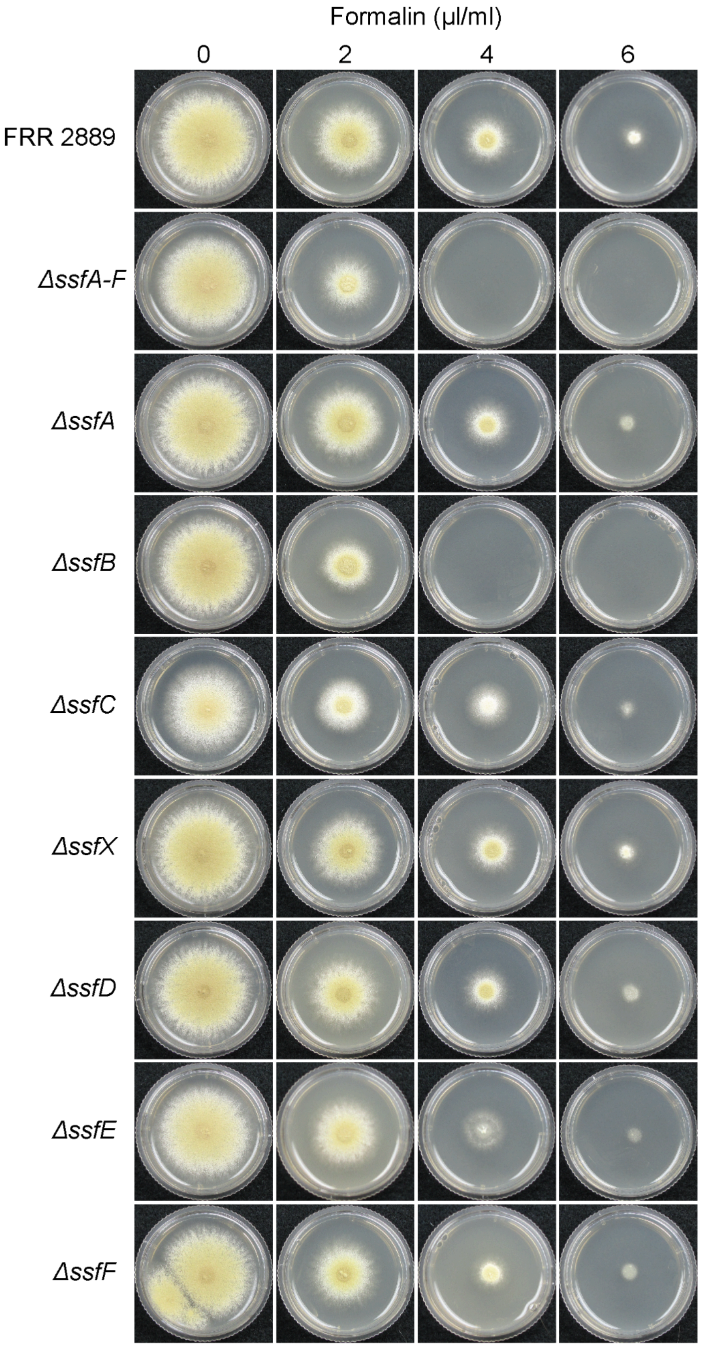

Figure S8: Deletion of different *ssf* genes had varying effects of formaldehyde resistance. Wild type *P. variotii* FRR 2889 and 8 *ssf* gene mutant strains grown on varying concentrations of formaldehyde for 3 days at 30°C.

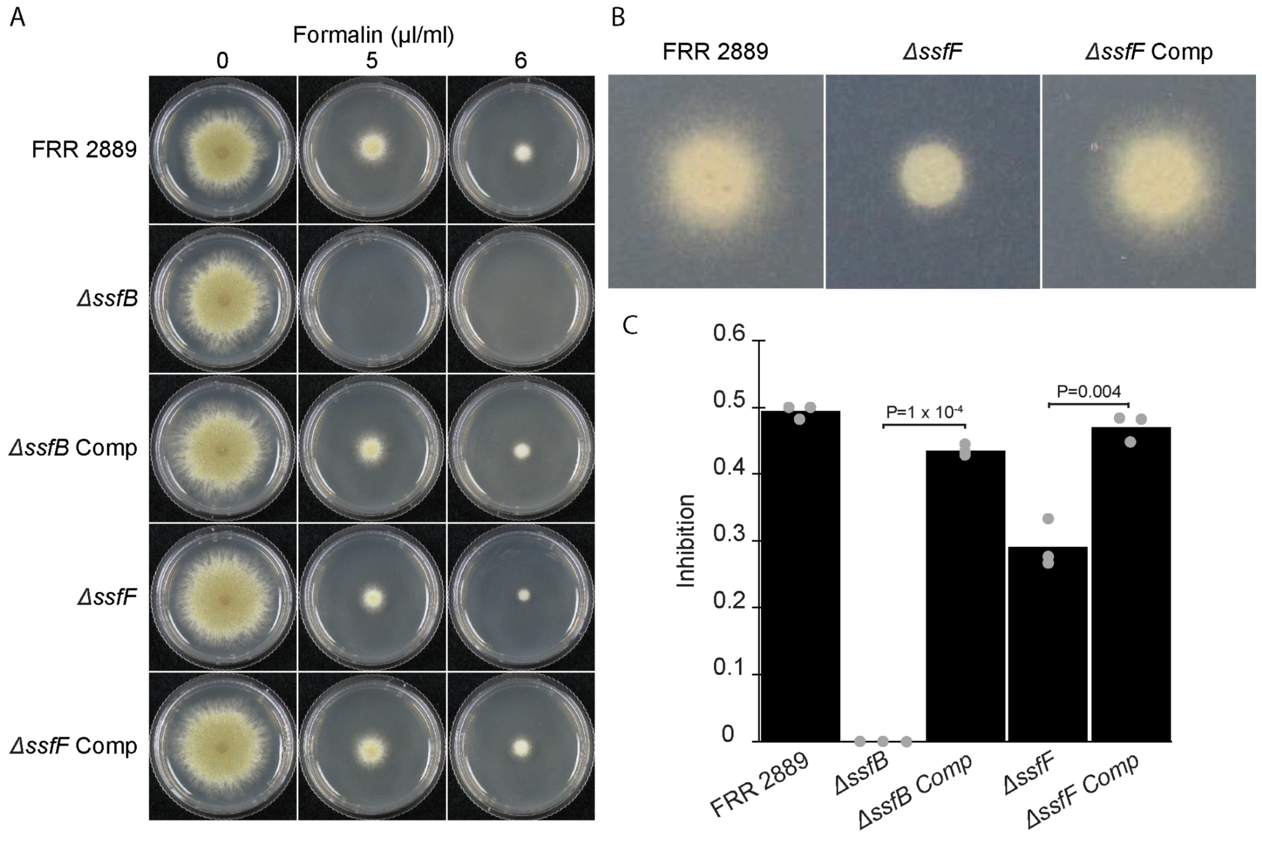

Figure S9: The *ΔssfB* and *ΔssfF* mutants were genetically complemented by addition of the corresponding wildtype gene. A) Growth of the *ΔssfB* and *ΔssfF* mutants compared to the wild type and to their genetically complemented counterparts (*ΔssfB* Comp and *ΔssfF* Comp) after 3 days at 30°C on different concentrations of formaldehyde. B) Magnified view of *ΔssfF* and *ΔssfF* Comp from panel A on 6 µl/ml formalin. C) Inhibition of radial growth by 4 µl/ml formalin compared to media without formalin after 3 days at 30°C.

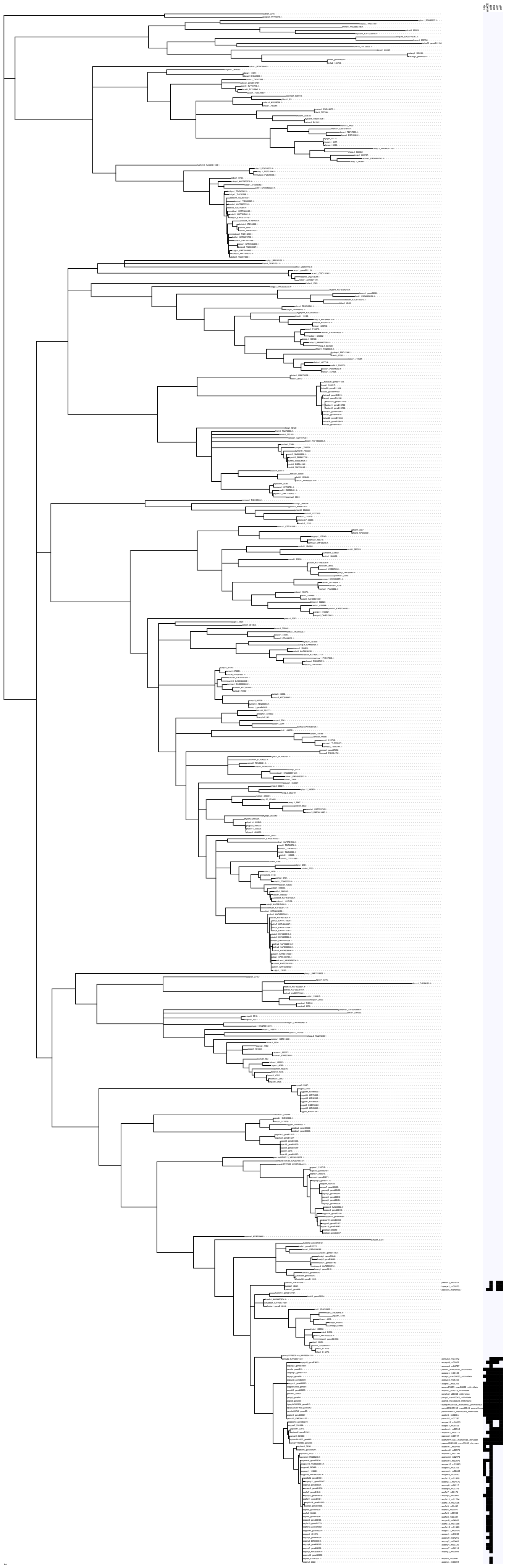

Figure S10: *SsfB* homologs found with *ssf* gene clusters are phylogenetically grouped. A midpoint-rooted maximum likelihood phylogeny of 468 *SsfB* sequences retrieved from the 2,899 genome database. Branch support was assessed with 1000 SH-aLRT tests and 1000 UFboot replicates. Branches with SH-aLRT support < 80% and UFboot support < 95% have been collapsed. *Starship* and gene neighborhood identifier codes are displayed to the right of sequences found in that associated region, for all sequences found to be a part of either a gene neighborhood or *Starship*. Neighborhoods were defined as containing homologs to at least 2 *ssf* genes of interest and *Starships* were either manually annotated or retrieved from the *Starship* database (Methods). To the right of the tree is a heatmap displaying the presence/absence of homologs to *Starship*- and *ssf*-associated genes in the *Starships* and gene neighborhoods associated with each sequence.

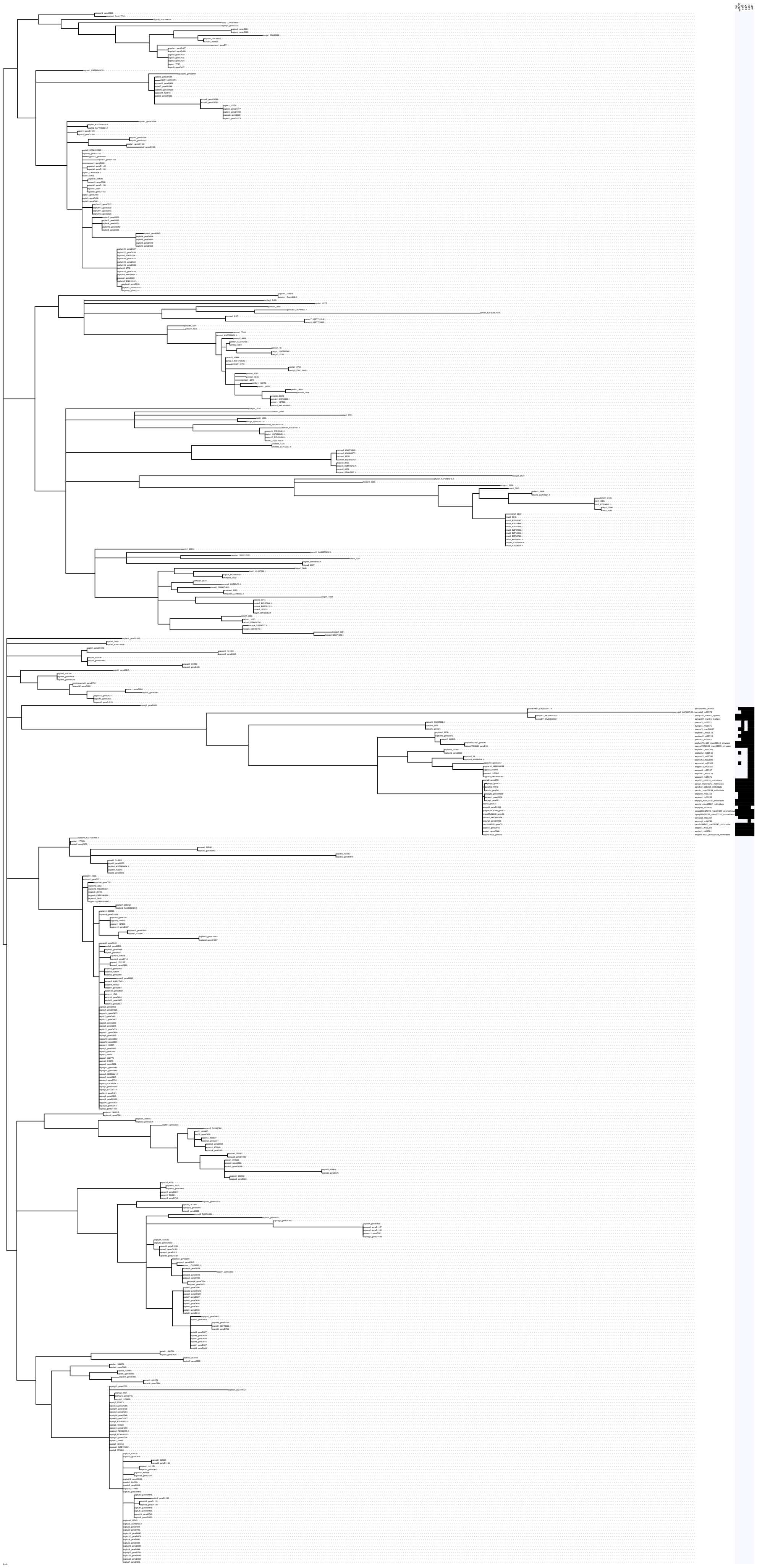

Figure S11: *SsfD* homologs found with *ssf* gene clusters are phylogenetically grouped. A midpoint-rooted maximum likelihood phylogeny of 484 *SsfD* sequences retrieved from the 2,899 genome database. Branch support was assessed with 1000 SH-aLRT tests and 1000 UFboot replicates. Branches with SH-aLRT support < 80% and UFboot support < 95% have been collapsed. *Starship* and gene neighborhood identifier codes are displayed to the right of sequences found in that associated region, for all sequences found to be a part of either a gene neighborhood or *Starship*. Neighborhoods were defined as containing homologs to at least 2 *ssf* genes of interest and *Starships* were either manually annotated or retrieved from the *Starship* database (Methods). To the right of the tree is a heatmap displaying the presence/absence of homologs to *Starship*- and *ssf*-associated genes in the *Starships* and gene neighborhoods associated with each sequence.

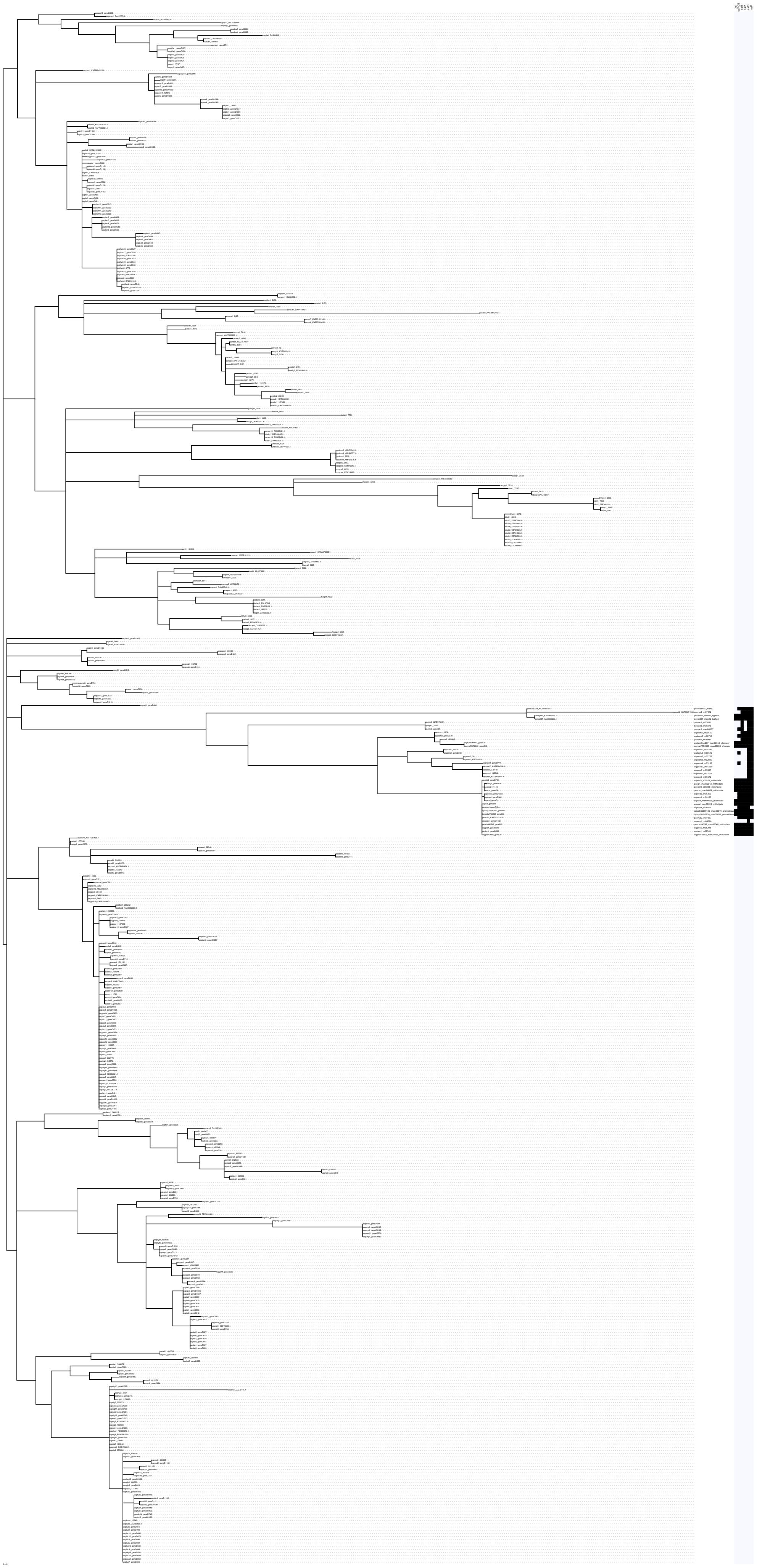

Figure S12: *SsfF* homologs found with *ssf* gene clusters are phylogenetically grouped. A midpoint-rooted maximum likelihood phylogeny of 124 *SsfF* sequences retrieved from the 2,899 genome database. Branch support was assessed with 1000 SH-aLRT tests and 1000 UFboot replicates. Branches with SH-aLRT support < 80% and UFboot support < 95% have been collapsed. *Starship* and gene neighborhood identifier codes are displayed to the right of sequences found in that associated region, for all sequences found to be a part of either a gene neighborhood or *Starship*. Neighborhoods were defined as containing homologs to at least 2 *ssf* genes of interest and *Starships* were either manually annotated or retrieved from the *Starship* database (Methods). To the right of the tree is a heatmap displaying the presence/absence of homologs to *Starship*- and *ssf*-associated genes in the *Starships* and gene neighborhoods associated with each sequence.

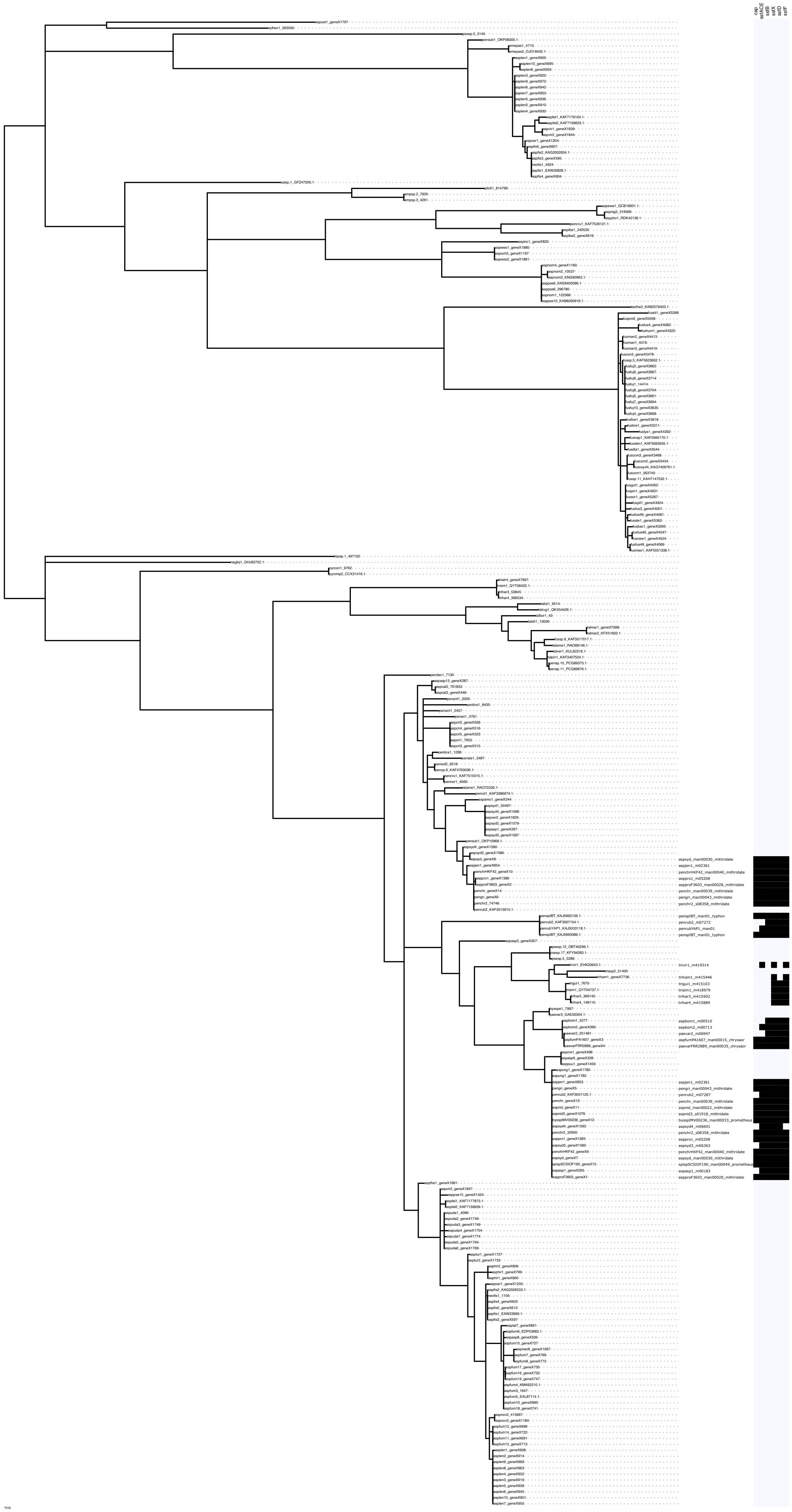
cap
ssfACE
ssfB
ssfX
ssfD
ssfF
aspsyd_man00030_mithridate
aspjen1_m02361
penchrHKF42_man00040_mithridate
asppro1_m05208
aspproF3603_man00028_mithridate
penchr_man00039_mithridate
pengri_man00043_mithridate
penchr2_s08358_mithridate
penspIBT_man01_typhon
penrub2_m07272
penrubYAP1_man01
penspIBT_man01_typhon
trivir1_m419314
triham1_m415446
trigui1_m415103
trisim1_m418979
trihar3_m415502
trihar4_m415889
aspbom1_m00510
aspbom2_m00713
paevar2_m06947
aspfumPA1607_man00015_chrysaor
paevarFRR2889_man00035_chrysaor
aspjen1_m02361
pengri_man00043_mithridate
penrub2_m07287
penchr_man00039_mithridate
aspnid_man00022_mithridate
aspnid3_s01918_mithridate
bysspIMV00236_man00033_prometheus
aspsyd4_m06601
penchr2_s08358_mithridate
asppro1_m05208
aspsyd3_m06363
penchrHKF42_man00040_mithridate
aspsyd_man00030_mithridate
spispSCSIOF190_man00049_prometheus
aspasp1_m00183
aspproF3603_man00028_mithridate

Figure S13: *SsfX* homologs found with *ssf* gene clusters are phylogenetically grouped. A midpoint-rooted maximum likelihood phylogeny of 248 *SsfX* sequences retrieved from the 2,899 genome database. Branch support was assessed with 1000 SH-aLRT tests and 1000 UFboot replicates. Branches with SH-aLRT support < 80% and UFboot support < 95% have been collapsed. *Starship* and gene neighborhood identifier codes are displayed to the right of sequences found in that associated region, for all sequences found to be a part of either a gene neighborhood or *Starship*. Neighborhoods were defined as containing homologs to at least 2 *ssf* genes of interest and *Starships* were either manually annotated or retrieved from the *Starship* database (Methods). To the right of the tree is a heatmap displaying the presence/absence of homologs to *Starship*- and *ssf*-associated genes in the *Starships* and gene neighborhoods associated with each sequence.

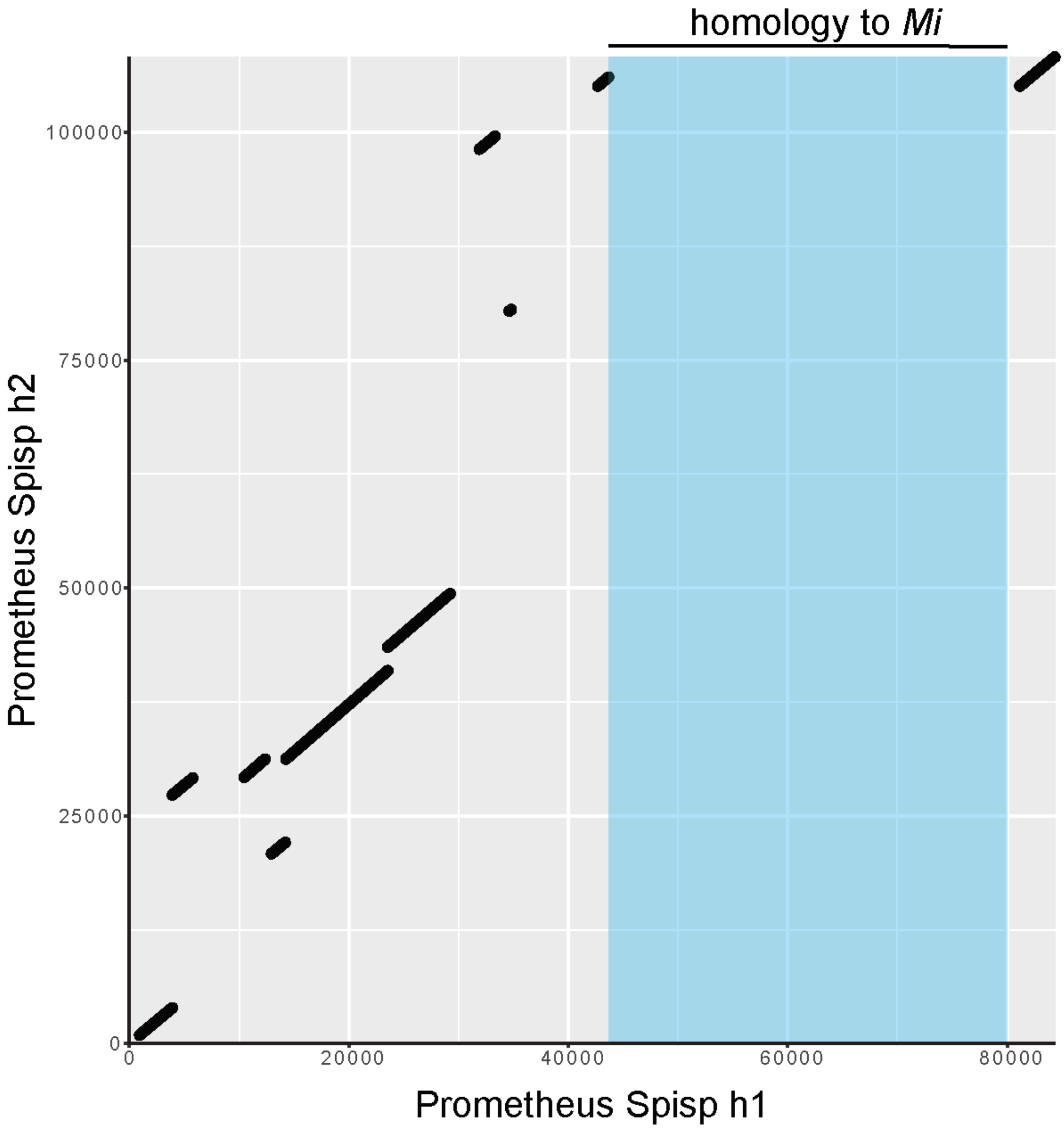

Figure S14: *Spiromastix sp*. SCSIOF190 contains two copies of *Prometheus* (*Πρ*), one that contains the *ssf* cluster and one that does not. Dot plot comparing the two *Πρ* haplotypes in *Spiromastix sp*. SCSIOF190. Only haplotype 1 contains a large region of homology to *Mithridate* (*Mi*) including the *ssf* cluster, this region is shaded blue.

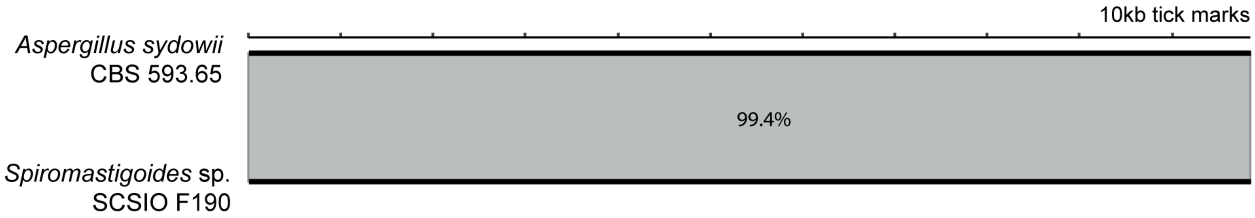

Figure S15: Comparison of *Prometheus* (*Пρ*) haplotype 2 (lacking the *ssf* cluster) *Starships* from *A. sydowii* CBS 593.65 and *Spiromastigoides sp.* SCSIO F190 shows them to be nearly identical. Nucleotide identity is given in percentages based on Mauve alignments.

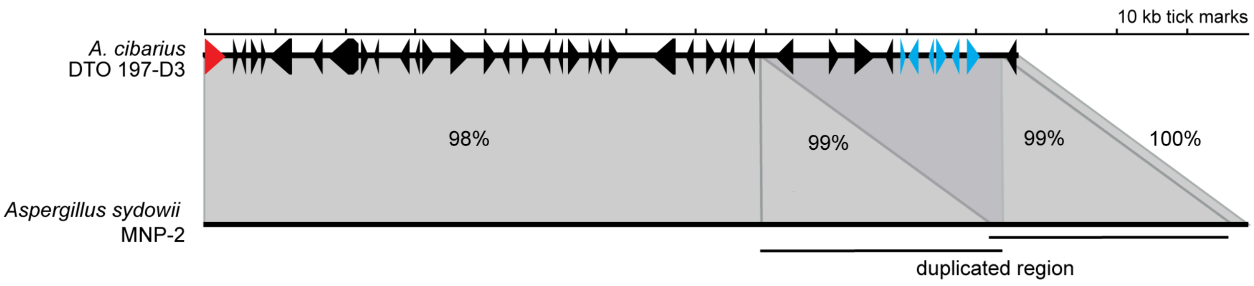

Figure S16: *A. cibarius* DTO 197-D3 and *A. sydowii* MNP-2 contain near identical *Prometheus* (*Πρ*) ssf *Starships*. Comparison of the *Πρ ssf Starships* from *A. cibarius* DTO 197-D3. Location: scaffold_21:162941-278881 against *A. sydowii* MNP-2, location: JAQTHS010000008.1:602259-453364. Nucleotide identity is given in percentages based on Mauve alignments. Gene models as depicted in Figure 1.

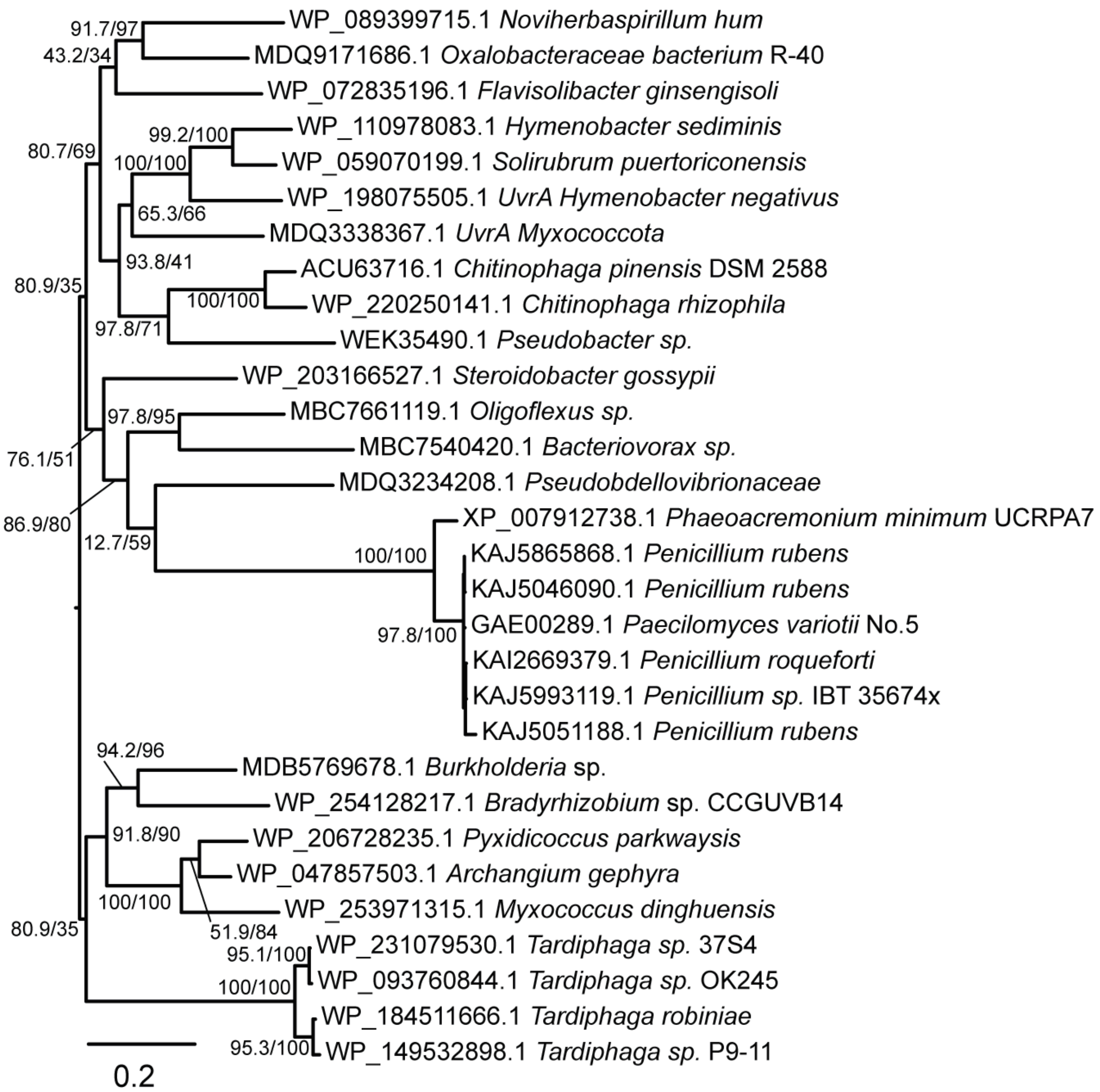

Figure S17: A protein phylogeny of a selection of closest homologs to the *UvrA* homolog encoded in certain *ssf* cluster-containing *Starships* shows close relationships to bacterial homologs. Sequences were aligned in MAFFT and tree generated using the default settings of IQ-TREE.

Supplementary Tables

Table S1: Colony diameters (mm) after 3 days growth on media with or without formaldehyde.

	FRR2889		ΔssfA		ΔssfB		ΔssfC		ΔssfD		ΔssfE		Δssf A-F		ΔssfF		ΔssfX	
**Formaldehyde**	**+**	**-**	**+**	**-**	**+**	**-**	**+**	**-**	**+**	**-**	**+**	**-**	**+**	**-**	**+**	**-**	**+**	**-**
Replicate 1	19	42	13	39	0	42	11	26	15	39	15	39	0	25	8	40	18	39
Replicate 2	18	41	16	42	0	42	12	26	15	39	15	40	0	28	8	40	19	40
Replicate 3	18	42	15	38	0	40	11	28	18	42	18	40	0	25	11	38	16	38
Replicate 4	19	38	14	40	0	40	12	25	16	38	17	39	0	22	10	39	18	40

Table S2: Colony diameters (mm) after 3 days growth on media with or without formaldehyde.

	FRR2889		ΔssfB		ΔssfB Comp		ΔssfF		ΔssfF Comp	
**Formaldehyde**	**+**	**-**	**+**	**-**	**+**	**-**	**+**	**-**	**+**	**-**
Replicate 1	14	28	0	25	12	27	9	27	13	27
Replicate 2	13	27	0	26	12	28	8	30	13	29
Replicate 3	13	26	0	25	13	30	8	29	14	29

Table S3: Colony diameters (mm) after 3 days growth on media with or without formaldehyde.

	FRR2889		ΔssfD		ΔpsfD		ΔssfD/ ΔpsfD		ΔssfF		ΔpsfF		ΔssfF/ ΔpsfF	
**Formaldehyde**	**+**	**-**	**+**	**-**	**+**	**-**	**+**	**-**	**+**	**-**	**+**	**-**	**+**	**-**
Replicate 1	20	33	18	40	11	20	0	38	14	35	17	33	7	34
Replicate 2	20	41	18	37	11	21	0	37	13	36	17	35	9	34
Replicate 3	18	35	18	37	12	20	0	35	13	35	15	35	7	36

Table S4: Genome database metadata. Data in supplemental file.

Table S5: Genomic coordinates of regions and *Starships* of interest. Data in supplemental file.

Table S6: Filtered BLAST hits using the LINE element present in *Prometheus* (*Πρ*) against assemblies of *A. sydowii* in Genbank as well as *Spiromastigoides sp. SCSIO F190*. Hits were filtered based on the following criteria: Percent identity >80%, start of hit within first 10% of query, length of hit > 10% of query.

Species and strain	Scaffold Accession	% identity	align ment length	misma tches	gap opens	query start	query end	subject start	subject end	evalue	bit score
Aspergillus sydowii AS31	JAIOTV0100 00164.1	95.378	5322	246	0	3	5324	31866	26545	0	8489
Aspergillus sydowii AS31	JAIOTV0100 00316.1	86.683	1209	161	0	4	1212	1	1209	0	1455
Aspergillus sydowii AS31	JAIOTV0100 00438.1	98.205	780	14	0	1	780	780	1	0	1344
Aspergillus sydowii AS42	JAIOTW0100 00021.1	95.378	5322	246	0	3	5324	56801	51480	0	8489
Aspergillus sydowii MNP-2	JAQTHS0100 00008.1	84.578	5317	810	5	4	5319	360617	365924	0	5879
Aspergillus sydowii MNP-2	JAQTHS0100 00008.1	83.356	727	118	3	18	744	354324	353601	0	755
Aspergillus sydowii Fsh102	SMUT010000 71.1	98.873	5324	60	0	1	5324	68892	63569	0	9331
Aspergillus sydowii Fsh102	SMUT010001 84.1	84.559	5317	811	5	4	5319	21396	16089	0	5874

Aspergillus sydowii Fsh102	SMUT01000184.1	82.944	727	121	3	18	744	27689	28412	0	742
Aspergillus sydowii Fsh102	SMUT01000483.1	99.371	1272	7	1	1	1272	1271	1	0	2255
Spiromastix sp. SCSIO F190	WXTY01000006.1	100	5382	0	0	1	5382	1492352	1497733	0	9707

Table S7: Primer sequences used to construct GFP replacement, knockout and complementation constructs used to genetically manipulate *Paecilomyces*.

Construct	Amplicon	Primer 1	Primer 2	Template
ssfA-F GFP replacement	5´ flank of *ssfF*	AUB656	AUB657	FRR 2889 gDNA
	GFP-term-HYGR	AUB658	AUB608	PLAUB69 [29]
	3´ flank of *ssfA*	AUB609	AUB610	FRR 2889 gDNA
ssfA GFP replacement	5´ flank of gene	AUB605	AUB606	FRR 2889 gDNA
	GFP-term-HYGR	AUB607	AUB608	PLAUB69 [29]
	3´ flank of gene	AUB609	AUB610	FRR 2889 gDNA
ssfB GFP replacement	5´ flank of gene	AUB611	AUB612	FRR 2889 gDNA
	GFP-term-HYGR	AUB613	AUB614	PLAUB69 [29]
	3´ flank of gene	AUB615	AUB616	FRR 2889 gDNA
ssfC GFP replacement	5´ flank of gene	AUB617	AUB618	FRR 2889 gDNA
	GFP-term-HYGR	AUB619	AUB620	PLAUB69 [29]
	3´ flank of gene	AUB621	AUB622	FRR 2889 gDNA
ssfD GFP replacement	5´ flank of gene	AUB623	AUB624	FRR 2889 gDNA
	GFP-term-HYGR	AUB625	AUB626	PLAUB69 [29]
	3´ flank of gene	AUB627	AUB628	FRR 2889 gDNA
ssfE GFP replacement	5´ flank of gene	AUB629	AUB630	FRR 2889 gDNA
	GFP-term-HYGR	AUB631	AUB632	PLAUB69 [29]
	3´ flank of gene	AUB633	AUB634	FRR 2889 gDNA
ssfF GFP replacement	5´ flank of gene	AUB656	AUB657	FRR 2889 gDNA
	GFP-term-HYGR	AUB658	AUB659	PLAUB69 [29]
	3´ flank of gene	AUB660	AUB661	FRR 2889 gDNA
ssfX GFP replacement	5´ flank of gene	AUB727	AUB728	FRR 2889 gDNA
	GFP-term-HYGR	AUB729	AUB730	PLAUB69 [29]
	3´ flank of gene	AUB731	AUB732	FRR 2889 gDNA

psfD Knockout	5´ flank of gene	AUB701	AUB702	FRR 2889 gDNA
	G418R	AUB703	AUB704	pMAI2 [96]
	3´ flank of gene	AUB705	AUB706	FRR 2889 gDNA
psfF Knockout	5´ flank of gene	AUB707	AUB708	FRR 2889 gDNA
	G418R	AUB709	AUB710	pMAI2 [96]
	3´ flank of gene	AUB711	AUB712	FRR 2889 gDNA
ssfF comp	Wildtype copy of gene	AUB755	AUB756	FRR 2889 gDNA
ssfB comp	Wildtype copy of gene	AUB699	AUB700	FRR 2889 gDNA

Table S8: Primers used to confirm correct site-specific integration of constructs into the *Paecilomyces* genome.

Transformant	Upstream primer	Downstream primer
ssfA GFP replacement	AUB666	AUB667
ssfB GFP replacement	AUB670	AUB671
ssfC GFP replacement	AUB674	AUB675
ssfD GFP replacement	AUB678	AUB679
ssfE GFP replacement	AUB682	AUB683
ssfF GFP replacement	AUB686	AUB687
psfD Knockout	AUB719	AUB720
psfF Knockout	AUB723	AUB724
ssfX GFP replacement	AUB741	AUB681
